# Avatar Intervention in Virtual Reality for Cannabis Use Disorder in Individuals With Severe Mental Disorders: Results From a 1-Year, Single-Arm Clinical Trial

**DOI:** 10.2196/58499

**Published:** 2024-11-27

**Authors:** Sabrina Giguere, Mélissa Beaudoin, Laura Dellazizzo, Kingsada Phraxayavong, Stéphane Potvin, Alexandre Dumais

**Affiliations:** 1 Department of Psychiatry and Addictology, University of Montreal, 2900 boulevard Édouard-Montpetit, Montreal, QC, H3T 1J4, Canada; 2 Research Center of the University Institute in Mental Health of Montreal, Montreal, QC, Canada; 3 Faculty of Medicine and Health Sciences, McGill University, Montreal, QC, Canada; 4 Services et Recherches Psychiatriques AD, Montreal, QC, Canada; 5 Institut National de Psychiatrie Légale Philippe-Pinel, Montreal, QC, Canada

**Keywords:** cannabis use disorder, cannabis use, virtual reality therapy, virtual reality, addiction intervention, relational therapy, avatar, digital mental health

## Abstract

**Background:**

The dual diagnosis of cannabis use disorder (CUD) and severe mental disorder (SMD) results in clinically complex individuals. Cannabis use is known to have negative consequences on psychiatric symptoms, medication compliance, and disease prognosis. Moreover, the effectiveness of currently available psychotherapeutic treatments is limited in this population. In this context, our research team developed avatar intervention, an approach using virtual reality as a therapeutic tool to treat CUD in individuals with SMD.

**Objective:**

This pilot clinical trial aimed to evaluate, until the 1-year follow-up, the efficacy of avatar intervention for CUD among 32 participants with a dual diagnosis of SMD and CUD.

**Methods:**

Over the course of the 8 intervention sessions, participants were given the opportunity to enter a dialogue in virtual reality with an avatar representing a person with a significant role in their consumption, who was animated in real time by a therapist. The primary outcomes were the quantity of cannabis consumed and the frequency of use. Secondary outcomes included severity of problematic cannabis use, motivation for change, protective strategies for cannabis use, consequences of cannabis use, psychiatric symptoms, and quality of life. Changes in reported outcomes during the assessment periods before the intervention; postintervention; and 3, 6, and 12 months after the end of the intervention were assessed using a linear mixed-effects model.

**Results:**

Significant reductions were observed in the quantity of cannabis consumed, and these were maintained until the 12-month follow-up visit (*d*=0.804; *P*<.001; confirmed by urine quantification). Frequency of cannabis use showed a small significant reduction at the 3-month follow-up (*d*=0.384; *P*=.03). Moreover, improvements were observed in the severity of CUD, cannabis-related negative consequences, the motivation to change cannabis use, and the strategies used to mitigate harms related to cannabis use. Finally, moderate benefits were observed for quality of life and psychiatric symptoms.

**Conclusions:**

Overall, this unique intervention shows promising results that seem to be maintained up to 12 months after the end of the intervention. With the aim of overcoming the methodological limitations of a pilot study, a single-blind randomized controlled trial is currently underway to compare the avatar intervention for CUD with a conventional addiction intervention.

## Introduction

Although cannabis is widely used in the general population, the prevalence of cannabis use is higher among individuals with severe mental disorder (SMD; including chronic psychotic and mood disorders) [1,2]. Indeed, meta-analyses have shown that cannabis use disorder (CUD) is present in approximately 1 in 5 people with schizophrenia or bipolar disorder, and 1 in 9 people with major depression [3-5]. Several factors may account for this association. Patients with SMD are more likely to progress to CUD and usually have more difficulty in achieving abstinence [5-8]. With regard to the initiation of drug use, several studies have suggested a common genetic and neurobiological vulnerability between SMD and CUD [9-12]. Once initiated, cravings are higher in individuals with SMD [10,13-15]. Also, individuals with SMD may use cannabis as a coping mechanism to temper their symptoms [16-19], as well as to help them suppress negative emotions and stress [20,21]. Thus, in the absence of a better emotion regulation strategy, substance use can be used as an avoidance strategy since it could suppress distress and reduce the intensity of negative emotions [17,19,22]. Moreover, this population is more likely to endure negative consequences associated with cannabis use, including exacerbation of psychiatric symptoms, reduced adherence to treatment, and increased hospitalization rates [23-29]. Considering that cannabis is being legalized in multiple states in countries and that Δ-9-tetrahydrocannabinol (THC) levels are rising steadily while appearing to be correlated with progression to CUD [30,31], effective treatments are critically needed [32,33].

Although several molecules are currently being studied for the treatment of CUD (eg, nabilone, topiramate, gabapentin, and fatty acid amide hydrolase inhibitor), no pharmacological treatment has been approved due to the limited evidence of efficacy available [34-36]. Thus, psychosocial interventions remain the first line of treatment [34]. Available psychotherapeutic interventions such as cognitive behavioral therapy (CBT) and motivational interviewing appear to have at best a moderate effect on the general population [37,38].

These same interventions do not appear to be as effective in individuals with SMD. Indeed, CBT, cognitive therapy, motivational interviewing, CBT plus motivational interviewing, or other combinations (ie, relapse prevention) have been found to be ineffective when compared to less active interventions or treatment as usual [39-45]. Regarding the effects of the interventions alone (ie, within-group results), very few studies have reported such findings. Among these, CBT, motivational interviewing, their combination, and treatment as usual have been shown to reduce the frequency or quantity of cannabis use [40,41,44]. However, specific recommendations regarding which type of intervention is more efficacious cannot be made due to the lack of evidence [46-48]. Hence, there is an urgent need to develop new treatment modalities adapted to this complex population and whose benefits would be maintained [49,50].

In recent years, virtual reality (VR) has emerged as a new modality for psychiatric interventions. Indeed, VR interventions allow patients to be immersed gradually in fully personalized and safe environments controlled by the therapist, recreating real situations in which it is possible to interact [51-54] and experience emotions similar to those experienced in life [55,56]. Also, VR overcomes certain limitations of current exposure therapies, where it may not necessarily be safe to expose patients in real life to certain environments and where there may lack professional resources [55]. Finally, VR allows patients to learn and apply strategies in real time, as the emotions generated during the immersion are similar in intensity to those experienced in everyday life, enabling better transposition [57,58]. VR has been used as a therapeutic tool to treat numerous psychiatric symptoms and disorders, such as psychotic disorders, anxiety disorders, obsessive-compulsive disorders, and eating disorders [54,58,59]. Regarding substance use disorders (SUDs), the efficacy of such therapeutic options has only been evaluated for nicotine dependence. While simple exposure to the substance in VR was not sufficient to obtain significant results, using VR as an opportunity to learn during immersion showed significant positive effects [60-62]. One crucial limitation common to all currently available VR interventions for SUDs remains that the environments used are predefined situations and dialogues are not in real time with an avatar. The scenarios could consequently not be personalized to the patient’s needs.

To improve the effectiveness of CUD treatments for people with SMD, our research team has developed the avatar intervention for CUD. To the best of our knowledge, this is the first intervention for the treatment of CUD focusing on relational components contributing to substance use while using VR as a personalized therapeutic tool. Results from an initial pilot study showed positive short-term benefits of this avatar intervention on cannabis use [63]. Indeed, results showed significant decreases in the quantity of cannabis consumed, as well as in the severity of CUD. The aim of this study was to evaluate the effects of the avatar intervention at 3, 6, and 12 months after the end of the intervention.

## Methods

### Participants

**Figure 1.**
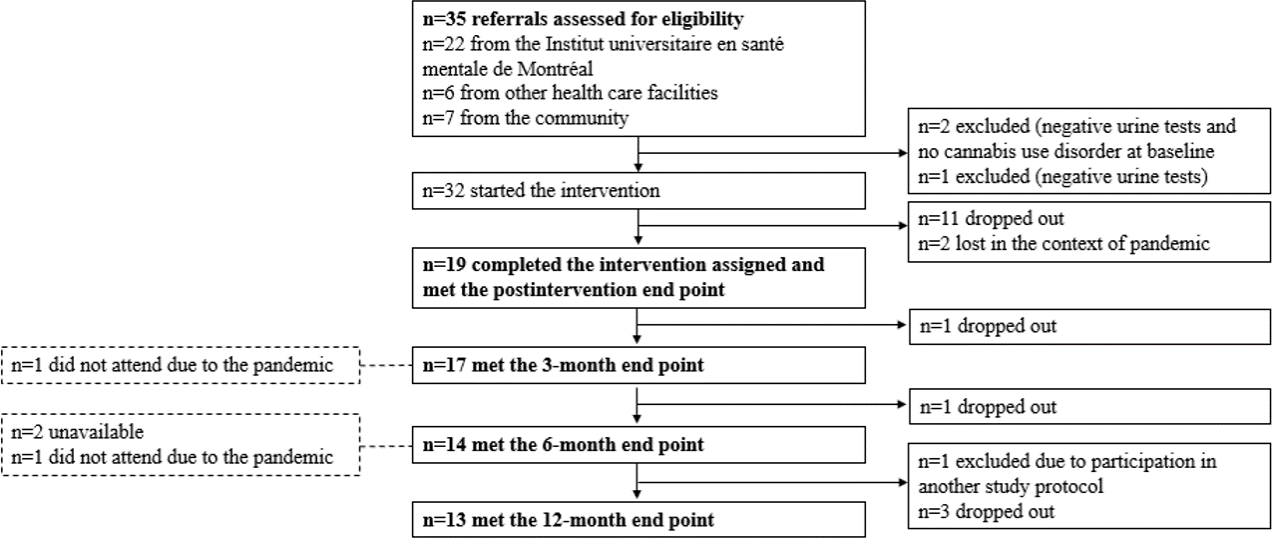
Trial profile of participants who underwent avatar intervention for CUD. There were 35 referrals provided by clinical teams and the community, 32 of whom were eligible. CUD: cannabis use disorder. Note: the dotted boxes on the left are participants who missed a follow-up, but returned at the next follow-up.

Participants were eligible if they were 18 years of age or older and had a diagnosis of moderate or severe CUD, as well as a diagnosis of a schizophrenia-spectrum disorder (ie, schizophrenia, schizoaffective disorder, and unspecified schizophrenia spectrum) or a chronic mood disorder (ie, bipolar disorder and major depressive disorder). The diagnoses were confirmed using the structured clinical interview for the *Diagnostic and Statistical Manual of Mental Disorders, Fifth Edition* (*DSM-5*) [64]. Participants were excluded if they had a neurological disease or an unstable serious physical illness. A total of 35 participants (32 outpatients and 3 inpatients) were recruited either from a psychiatric hospital (Institut universitaire en santé mentale de Montréal) or from the community (Figure 1). The study took place from October 2019 to May 2023.

### Ethical Considerations

The trial was approved by the ethics committee of the Centre intégré universitaire de santé et de services sociaux de l’Est-de-l’Île-de-Montréal (CEMTL; 2019-1564) and all participants provided informed written consent. Participants received a compensation amount of CAD $20 (CAD $1 was equivalent to US $0.74) for each assessment. Data were collected at the Centre intégré universitaire de santé et de services sociaux de l’Est-de-l’Île-de-Montréal and were anonymized.

### Design

This single-arm trial is part of the clinical trial (ClinicalTrials.gov; NCT05726617) [63]. Clinical evaluations were performed in person by a trained psychiatric nurse about 1 week before the beginning of the intervention (baseline) and 1 week after the intervention (postintervention), as well as 3, 6, and 12 months after the end of the intervention. To comply with the time frames for the follow-up period, assessments had to be carried out within a maximum of 1 month to be included. Participants continued to receive their standard psychiatric care throughout the study. This paper presents the results of the medium- and long-term follow-ups (3, 6, and 12 months) of the previously published pilot clinical trial (comprising only results of the postintervention) [63].

### Avatar Intervention

#### Overview

Participants attended at least 8 weekly sessions (mean 10.2, SD 2.97) lasting between 60 and 90 minutes. The intervention was provided by an experienced psychiatrist (AD) with more than 10 years of experience working with this complex population. With the aim of gaining greater control over their cannabis use, the avatar intervention for CUD exposed immersed participants to the substance; to situations at risk of use; and to contexts that generate cravings, negative emotions (eg, anger, sadness, disappointment, and shame), and stressors that can precipitate use. To achieve this, participants create avatars representing significant people associated with their substance use, who will be played by the therapist. The therapist can play the role of a drug dealer, family member or friend, depending on which avatar the patient wishes to interact with at each session. The therapist who plays the role of each avatar during the sessions uses the same vocabulary and expressions as the person represented by the avatar. For example, if the avatar represents a consumer friend, 1 of the strategies was to encourage participants to find alternatives to consumption (eg, avatar: “We should do something together other than consume, what should we do?”). Avatars were created by the participants, with assistance as needed, based on physical characteristics (eg, facial features; eye, hair, and skin color; haircut, and so forth). In this way, the personification of avatars and the relational component established between the avatar and the patient allowed to generate personalized cravings and emotions for each participant. The aim was for the interactions and the immersive context to be as representative as possible of each participant’s reality. The immersive sessions were separated into 3 phases, each lasting around 20 to 30 minutes.

#### Preimmersion

Preimmersion is the summary of the preceding week and the determination of the objectives of this intervention session. The participant and the therapist decided face-to-face together what the aim of the scenario would be. They also decided which avatar would be selected as well as the VR environment (in a bar, an apartment, or a park).

#### Immersion

**Figure 2.**
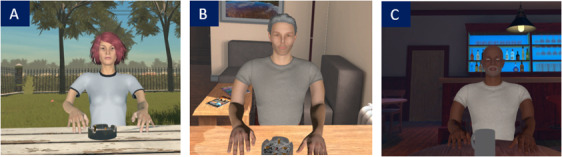
Example of avatars in VR environment. (A) Park, (**B**) apartment, and (C) bar. VR: virtual reality

**Figure 3.**
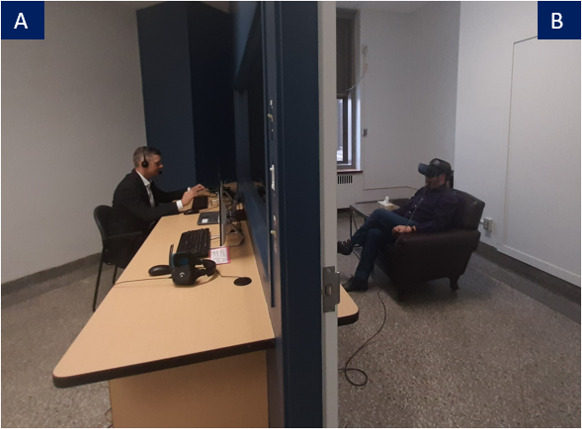
**(A**) Therapist’s side and (**B**) participant’s side. The participant, wearing a VR headset and noise-cancelling headphones, sat in an adjacent room and was invited to enter a dialogue with their avatar in the VR environment. The therapist could see the participant through a 1-way mirror. Explicit consent was obtained from the individuals in this figure to use their image for publication. VR: virtual reality.

In immersion, the therapist animated the avatar by having his voice modified in real time, as well as by controlling the avatar’s facial expression (ie, angry, sad, joyful, and fearful). During these interactions, participants were encouraged to practice their coping mechanisms and interpersonal skills (eg, self-affirmation). Please see Figure 2 for examples of avatars and environment and Figure 3 for a visual of the setting in which immersion takes place.

#### Postimmersion

Postimmersion is the debriefing of the participant’s experience, including the feelings that arose during the immersion.

The interactions between the participants and their avatars allowed them to practice coping strategies in real time. Changes in relationships with cannabis, others, and oneself were at the core of each session. The intervention, including the materials and course of the sessions, was described in further detail elsewhere [63,65].

### Clinical Assessments

The primary outcomes were the quantity and the frequency of cannabis consumption, which were both assessed using the Timeline Follow-Back (TLFB). The TLFB is a self-reported questionnaire evaluating substance use in the previous week (eg, cannabis, alcohol, and amphetamines) and has strong interrater reliability [66,67]. For cannabis use, the number of joints was reported, as equivalent to 0.5 g each. Moreover, to confirm the self-report data on the amounts of cannabis consumed by the participants, urine levels of 11-nor-9-Carboxy-Δ9-tetrahydrocannabinol (THC-COOH) were quantified before the intervention and after the intervention, as well as at the 3-months follow-up [68]. The detailed methodology of that test has been described elsewhere [63]. To verify that cannabis had not been replaced by another substance, the consumption of alcohol and other recreational drugs was also monitored. To have a comparable unit of measurement, all quantities were converted to sale prices as reported by participants. Consequently, a nonsignificant result would indicate that there is no increase in the consumption of other drugs.

Secondary outcomes included the severity of problematic cannabis use, motivation for change, protective strategies for cannabis use, consequences of cannabis use, psychiatric symptoms, and quality of life. Problematic cannabis use was assessed using the Cannabis Use Problems Identification Test (CUPIT), a self-reported questionnaire with good to excellent test-retest reliability and internal consistency [69]. The marijuana ladder was used to measure participants’ motivation to change their cannabis use habits. This tool has a good concurrent and predictive validity assessing their readiness to change (precontemplation, contemplation, preparation, action, and maintenance) [70]. The strategies used to mitigate harms related to cannabis use were measured by the Protective Behavioral Strategies for Marijuana Short Form (PBSM-SF), which demonstrated satisfactory fidelity, construct validity, and criterion-related validity [71,72]. The negative consequences associated with cannabis use were assessed with the Brief-Marijuana Consequences Questionnaire (B-MACQ) [73]. Quality and satisfaction of life were assessed using the Heinrichs’ Quality of Life Scale (QLS), a 21-item tool covering different spheres of quality of life [74]. The Positive and Negative Syndrome Scale (PANSS) was used to evaluate psychiatric symptoms. The PANSS was shown to have good interrater reliability, an appropriate test-retest reliability, and a high internal consistency [75,76]. Evaluators were trained to administer this scale, and interrater reliability was ensured by conducting consensus ratings on standardized videotapes and real patients. Because a change in pharmacological treatment during the intervention could potentially impact psychiatric symptoms, the participants’ medication lists before and after the therapy were compared. As no significant change was observed, this variable was not considered in further analyses.

### Statistical Analysis

Statistical analyses were performed with SPSS (version 28; IBM Corp). Changes in reported outcomes during the assessment periods before the intervention; postintervention; and 3, 6, and 12 months after the end of the intervention were assessed using a linear mixed-effects model with maximum likelihood estimations for missing data [77-79]. Of note, it was expected that the postintervention results might slightly differ from previously published results, as follow-up time points were also considered in the statistical model [63]. Quantification of THC-COOH in urine was performed by applying the creatinine concentration ratio. Changes in THC-COOH were assessed using Wilcoxon for paired variables. To assess whether participants who dropped out before the end of the protocol (12-month follow-up) had a particular profile, baseline sociodemographic, and clinical characteristics were analyzed. For continuous variables, a 2-tailed *t* test was performed for normally distributed data, and a Mann-Whitney test was performed for nonnormally distributed data. For dichotomous variables, a Pearson chi-square test was used to assess categorical outcome differences. In this case, 1 cell or more in the contingency table had a frequency under 5; the Fisher exact test was used, as it is more applicable to smaller samples [80]. Although the intervention was planned for 8 sessions, keeping in mind that this was a pilot project, the therapist remained flexible and adapted the number of sessions to each participant (range 8‐15). The association between the number of sessions and efficacy of primary outcomes (quantity and frequency of cannabis use) was, therefore, examined. To do so, Spearman correlations were calculated between the number of sessions and primary outcomes (ie, the variation in quantity and frequency of cannabis use). The following descriptions of the strength of reported Cohen *d* were used: small ]0.2‐0.5[, medium [0.5‐0.8[, and large ≥0.8 [81]. The statistical threshold for significance was set at *P*<.05.

## Results

### Sample Characteristics

Detailed sample characteristics can be found in Table 1. Participants were mostly men, Caucasian, and unemployed. Most participants had a primary diagnosis of a schizophrenia spectrum disorder. Moreover, most of the subsample had an antisocial or borderline personality disorder. The age ranged between 23 and 58 years. The average age of cannabis initiation was 14.7 years old. Almost all participants had a severe CUD. A significant proportion of participants had another SUD, notably alcohol and stimulants (Table 1).

**Table 1.** Baseline sociodemographic and clinical characteristics (n=32).

Characteristics	Values
Age (years), mean (SD)	36.6 (9.47)
**Sex, n (%)**	
Male	27 (84.4)
Female	5 (15.6)
**Ethnicity, n (%)**	
Caucasian	28 (87.5)
Other	4 (12.5)
Currently employed, n (%)	10 (31.3)
**Severity of cannabis use disorder, n (%)**	
Moderate	2 (6.3)
Severe	30 (93.7)
Age of onset of cannabis use, mean (SD)	14.7 (1.99)
**Other current substance use disorder, n (%)**	
Alcohol	11 (34.4)
Stimulants (cocaine, crack, and amphetamine)	18 (56.3)
Other^a^	4 (12.5)
**Dual diagnosis, n (%)**	
Psychotic disorder	25 (78.2)
Bipolar disorder	2 (6.2)
Major depressive disorder	5 (15.6)
**Comorbid personality disorders (n=19)** ^**b**^ **, n (%)**	
Antisocial	9 (47.4)
Borderline	7 (36.8)

a Other: sedatives, opiates, Gamma-hydroxybutyric acid (GHB), and ketamine.

b This measurement was added to baseline clinical evaluations later during the trial, and therefore, the first 13 participants were not assessed for personality disorders.

### Intervention Efficacy

#### Primary Outcome

The avatar intervention showed numerous benefits that persisted over time (Table 2). Indeed, a significant reduction in the quantity of cannabis consumed (joints) was observed at all time points. Indeed, improvements continued to be seen over time, and the largest effect size was observed at the 12-month follow-up visit (*d*=0.804; *P*<.001). The urinary THC-COOH quantification confirmed this reduction in 13 participants from whom urine samples were taken at baseline (mean 708.85, SD 1099.91), postintervention (mean 455.83, SD 622.47; *P*=.04), and 3-months follow-up (mean 252.61, SD 343.49; *P*=.02). Additionally, there was a strong correlation between THC-COOH quantification and self-reported amounts of cannabis (*r*=0.791; *P*<.001). A significant reduction in the frequency of cannabis use was observed at 3-month follow-up compared to baseline (*d*=0.384; *P*=.03); however, these improvements were no longer seen during the subsequent follow-ups. Cannabis use was not replaced by another substance as the amount of money spent on all other substances combined (alcohol and drugs, excluding cannabis) decreased from CAD $62 to CAD $13.20 at the 12-month follow-up.

**Table 2.** Change in clinical outcomes observed at the postintervention, 3, 6, and 12-month follow-up visits compared to baseline^a^.

	Baseline (n=32)	Postintervention (n=19)			3-month follow-up (n=17)			6-month follow-up (n=14)			12-month follow-up (n=13)		
	Mean (SD)	Mean (SD)	*P* value	Cohen *d*	Mean (SD)	*P* value	Cohen *d*	Mean (SD)	*P* value	Cohen *d*	Mean (SD)	*P* value	Cohen *d*
Cannabis quantity (number of joints in last week)	24.4 (27.5)	9.81 (19.6)	.001^b^	0.671^b^	13.2 (15.2)	.004^b^	0.513^b^	13.0 (22.1)	.003^b^	0.522^b^	6.90 (8.44)	<.001^b^	0.804^b^
Cannabis frequency (days in last week)	5.20 (2.70)	4.28 (3.24)	.05	0.320	4.08 (2.89)	.03^b^	0.384^b^	5.12 (2.97)	.83	0.028	4.87 (3.09)	.68	0.112
Money spent on drugs and alcohol excluding cannabis use (CAD $^c^ per week in last week)	62.0 (194)	15.5 (33.6)	.17	0.403	12.1 (32.7)	.13	0.432	12.8 (28.9)	.15	0.426	13.2 (26.5)	.14	0.422
Problematic cannabis use (score)	37.4 (10.87)	31.8 (12.4)	.02^b^	0.507^b^	31.2 (11.7)	.007^b^	0.562^b^	31.5 (9.03)	.005^b^	0.534^b^	31.2 (9.34)	.005^b^	0.558^b^
Motivation to change cannabis use (score)	6.09 (2.13)	7.55 (2.22)	.009^b^	0.584^b^	7.77 (2.34)	.01^b^	0.674^b^	6.64 (3.06)	.41	0.223	6.71 (3.02)	.38	0.248
Strategies used to mitigate harms related to cannabis use (score)	49.6 (12.9)	48.8 (15.0)	.80	0.050	60.2 (16.9)	.008^b^	0.593^b^	57.2 (24.1)	.03^b^	0.428^b^	63.3 (19.0)	<.001^b^	0.769^b^
Negative consequences associated with cannabis (score)	9.52 (4.82)	8.02 (4.97)	.17	0.294	7.86 (5.74)	.21	0.335	6.68 (5.12)	.01^b^	0.561^b^	7.19 (3.92)	.007^b^	0.461^b^
Quality and satisfaction of life (score)	59.5 (18.8)	63.9 (17.6)	.10	0.243	71.8 (15.0)	.004^b^	0.675^b^	67.1 (16.3)	.07	0.417	69.2 (20.5)	.03^b^	0.532^b^
Psychiatric symptomatology (score)	71.8 (12.0)	69.0 (16.1)	.22	0.308	63.7 (10.5)	.01^b^	0.640^b^	64.4 (9.95)	.03^b^	0.579^b^	61.8 (12.4)	.03^b^	0.782^b^
Positive symptoms (score)	17.6 (4.46)	17.2 (4.56)	.64	0.0830	15.8 (4.19)	.04^b^	0.417^b^	15.0 (3.88)	.06	0.585	16.1 (4.25)	.22	0.333
Negative symptoms (score)	18.7 (4.12)	18.1 (4.48)	.39	0.164	16.2 (3.42)	.02^b^	0.607^b^	17.2 (4.46)	.20	0.372	15.5 (4.15)	.05	0.788
General symptoms (score)	35.6 (6.61)	33.6 (8.28)	.25	0.308	32.1 (4.31)	.04^b^	0.524^b^	31.0 (4.98)	.02^b^	0.693^b^	29.8 (6.07)	.01^b^	0.878^b^

a Linear mixed models with maximum-likelihood estimation were used to estimate *P* values and effect sizes. Of note, it was expected that the postintervention results might slightly differ from previously published results, as follow-up time points were also considered in the statistical model [63].

b Statistically significant differences (*P*<.05).

c CAD $1 was equivalent to US $0.74.

#### Secondary Outcome

Regarding the severity of problematic cannabis use, a reduction corresponding to a moderate effect size was obtained at all time points. A moderate increase in motivation to change in cannabis use at postintervention and at 3-month follow-up was observed, but this increase in motivation did not remain significant at 6 and 12 months. Also, a significant improvement in the strategies used to mitigate harms related to cannabis use was observed at all times points. Indeed, improvements continued to be seen over time, and the largest effect size was observed at the 12-month follow-up visit (*d*=0.769; *P*<.001). A reduction in negative consequences associated with cannabis compared to the baseline was observed, which was significant from the 6-month follow-up. Participants experienced important improvement in quality of life, which was significant at 3 and 12 months (*d*=0.675; *P*=.004 and *d*=0.532; *P*=.04), and a nonsignificant trend in postintervention and 6 months. From the 3-month follow-up, moderate improvement in psychiatric symptoms was observed with psychiatric symptoms and continued to be seen over time. In particular, there was a significant reduction in positive and negative symptoms at the 3-month follow-up, as well as a reduction in general symptoms at all follow-ups. During the intervention, most participants did not sustain a change in their daily medication dosages (14/19, 73.7% of participants). Of the 19 participants who completed the intervention, 2 reduced their daily medication dose (25 mg of loxapine; 50 mg of quetiapine), 1 stopped his antidepressant medication (150 mg of bupropion XL), and 1 slightly increased his antidepressant dose (25 mg of sertraline). Finally, only 1 participant drastically changed their medication regimen following a psychotic decompensation that occurred during the course of the therapy and necessitated the addition of a second antipsychotic and a mood stabilizer.

Concerning the baseline profile of participants who completed the 12-month follow-up (n=13) compared to noncompleters (n=19), analyses showed no statistically significant sociodemographic or clinical between groups. At baseline, the amount and frequency of cannabis use, severity of CUD, and motivation to change cannabis use were not significant. However, an amphetamine use disorder was significantly associated with quitting (*P*=.04), while alcohol and cocaine use disorders were not. Finally, the number of sessions was not significantly correlated with the 2 main outcomes (reduction in quantity and frequency of cannabis use) at each follow-up when compared to baseline data.

Linear mixed models with maximum-likelihood estimation were used to estimate *P* values and effect sizes. Of note, it was expected that the postintervention results might slightly differ from previously published results, as follow-up time points were also considered in the statistical model [63].

## Discussion

### Principal Findings

In the context where there is a need for effective interventions with long-term benefits for CUD in a population with SMD, this study aimed to evaluate the effects of a novel VR intervention to treat CUD for up to 1 year following the end of the intervention. Although avatar intervention for CUD links several preexisting concepts (eg, motivational approach, cognitive-behavioral approach, and relapse prevention), its particularity is that it allows patients to practice their strategies in a safe environment where cravings and emotions are elicited in a similar intensity to that of their daily life. This intervention’s relational and immersive aspects, which are entirely personalized, are hypothesized to enable the transposition of learning into everyday life [57,58], leading to significant improvements. Indeed, results from this study show significant reductions in the total amount and frequency of cannabis use, which were maintained over time. Improvements were also noted in the severity of problematic cannabis use, negative consequences, motivation for change, psychotic symptoms, and quality of life.

Regarding the primary outcome, there was a significant decrease in the amount of cannabis use that was maintained until the 12-month follow-up visit and a significant decrease in the frequency at the 3-month follow-up. This result was also validated by a decrease in cannabis use as measured by quantitative urinary analyses. Moreover, cannabis consumption did not seem to have been replaced by other substances, as the total cost of drug acquisition remained lower at each time point when compared to the baseline. Although this is a pilot trial, these results are promising, as to the best of our knowledge, the existing interventions for CUD in a population with SMD generally show either no significant effects or, at best, small effects on cannabis use [39-45,45]. Indeed, there have been mixed results for cognitive behavioral–oriented interventions, motivational interviewing, the combination of both, and treatment as usual. In addition to practicing strategies in the VR environment, the relational approach is likely central to this intervention. Targeting interpersonal relationships, which are important factors influencing cannabis use, may be a key mechanism leading to potential benefits [82-84].

Regarding secondary outcomes, the severity of problematic cannabis use significantly decreased after the intervention, and these changes were maintained until the 12-month follow-up. Similarly, there was a significant reduction in the negative consequences associated with cannabis at the 6- and 12-month visits. Motivation for change initially improved but returned to baseline by the 6-month visit. Regarding strategies used to mitigate harms related to cannabis use, significant benefits were observed starting at 3 months and up to 12 months after the intervention. These results are in line with the literature on the subject. Indeed, when cannabis use is reduced, there is an improvement in the severity and the consequences associated with cannabis use [85]. Also, an increase in protective behavioral strategies was associated with fewer negative consequences associated with cannabis use [86]. Moreover, improving participants’ harm-reduction strategies associated with cannabis use is in line with the premise of the avatar intervention. As previously mentioned, using VR and a dialogical approach was hypothesized to allow a better transposition of the strategies into everyday life. A future study comparing a traditional face-to-face intervention to a VR intervention should evaluate whether the intensity of emotions contributes to the maintenance of acquired skills after the end of the intervention.

Interestingly, both quality of life and psychiatric symptoms, which had not significantly changed right after the intervention, showed significant improvements during the follow-up period. Considering that the postintervention assessment was administered within a week of the end of the intervention, it is likely that a delay between changes in consumption is necessary to achieve effects on quality of life and symptomatology. To the best of our knowledge, this is the first intervention for CUD to improve the quality of life and psychiatric symptoms in a population with SMD, and this was maintained up to 1 year after the end of the intervention. Although maintenance of medication during intervention was not an inclusion criterion in this pilot study, assessment of medication at baseline and postintervention showed little changes, which were not significant and thus not likely to influence the observed improvement in psychiatric symptoms. Nevertheless, significant changes were observed for 1 participant, which could have led to biased results. To overcome this potential limitation, an intention to maintain medication during intervention was added as an inclusion criterion in an ongoing randomized controlled trial. These results are partly in line with studies that have evaluated the evolution of psychiatric symptoms and quality of life in people with SMD over 1 year comparing three groups—(1) continued cannabis use, (2) discontinued use, and (3) no cannabis use [29,87]. Regarding psychiatric symptoms, a meta-analysis observed that persistent cannabis use had a greater increase symptoms compared to those who had stopped. Also, individuals who had stopped using cannabis did not have a significantly different risk from nonusers. In particular, the severity of positive symptoms is affected in persistent cannabis users compared to those who have stopped. However, continued cannabis use did not increase negative symptoms [29]. Although the reduction in cannabis use likely contributed to the observed improvements in psychiatric symptoms, it is also possible that improved emotion management and greater interpersonal skills, which were both learned and practiced during the intervention, could also take part in these benefits [88]. Concerning the quality of life, there did not appear to be any significant difference between individuals who continued to use, those who stopped, and nonusers [87]. These results suggest that cannabis reduction alone may not fully explain the changes observed. Indeed, VR therapy for another psychiatric condition with a relational component showed significantly improved quality of life [89]. Changes in relationships with cannabis, others and oneself were at the core of each session; therefore, it is not surprising that benefits were observed in participants’ quality of life. Indeed, by promoting alternatives to consumption, participants may have developed new activities. Also, having acquired conflict resolution skills, it is possible that relationships with others may have improved. As the specific mechanisms of this new intervention have yet to be elucidated in comparison with a conventional face-to-face intervention, these will need to be more deeply investigated in future studies.

Participants’ engagement, as well as the acceptability and feasibility of the intervention were thoroughly evaluated in a prior study [63]. First, in response to an initially very high attrition rate, highly motivated participants were subsequently preferred, which resulted in a drastic reduction in dropouts. The overall attrition rate during the treatment was 40.6%, which is similar to those observed in a population with an SMD [90,91]. Of the 19 participants who completed the postintervention evaluation, 4 additional participants dropped out before the last follow-up. Second, an analysis of baseline sociodemographic and clinical characteristics revealed that only amphetamine use disorder was associated with a reduced likelihood of completing the intervention and the follow-up. A meta-analysis likewise identified stimulant use disorder as an element influencing dropout rates in individuals with SMD [90]. To address potential selection bias, linear mixed models including the presence or absence of amphetamine use disorder as a covariable were calculated (data not shown), and the results remained grossly unchanged. Third, semistructured interviews were conducted until data saturation (n=11) to evaluate the participants’ satisfaction with each component of the intervention (eg content, frequency, number and sequence of sessions, appropriateness, and use of VR). Overall, the level of satisfaction was found to be high. Finally, adverse events were systematically recorded, showing that none of the participants discontinued [63]. Cannabis use can lead to increased hospitalization in our population, which is associated with substantial societal costs. Considering that, the costs associated with the acquisition of VR equipment and therapist training could be justified by a reduction in the use of health care services. Therefore, a cost-effectiveness analysis of VR use for CUD treatment in a population with SMD will be necessary prior to implementing this intervention in regular clinical practice [31,92,93]. Additionally, technological advances in recent years have made VR equipment more affordable, which could eventually make it easily available and accessible in clinics, especially since therapists appear to be open to its use [94,95]. Moving forward, it is foreseeable that most clinics and hospitals will make VR equipment available for therapists wishing to deliver various evidence-based interventions using this technology.

While the results suggest that the avatar intervention for CUD appears to be a promising avenue in individuals with SMD and CUD, the present trial has some important limitations to highlight. First, this study does not include a control group for comparison purposes. Considering studies have observed a regression toward the mean of psychiatric symptoms, naturally, in assessments with several time points as well as to control the nonspecific effects of psychotherapies, a control group intervention will be necessary in future clinical trials [96,97]. Second, as there was no control group, the evaluators could not be blind to the intervention. Third, as this was a pilot trial, the sample size was small. To address these three major limitations, a single-blind randomized controlled trial with a projected enrollment of 150 participants comparing the avatar intervention for CUD with a conventional addiction intervention is underway. Fourth, the THC/CBD ratio was not considered, mainly because participants generally did not know exactly what they were consuming. A potential solution to this problem would be to analyze a sample of each participant’s cannabis; however, this raises ethical and legal issues that could be complex to resolve. Fifth, in this first clinical pilot, the interventions were offered by a single therapist. In the future, it is planned to integrate other service providers and to harmonize the intervention by means of training and an intervention manual. Finally, in the interests of desirability, patients may have underestimated their consumption. However, quantitative analyses of THC-COOH undertaken at baseline, postintervention, and the 3-month follow-up show a strong correlation with patients’ self-reported measures (as observed in other studies using similar quantification methods), indicating that they have a good assessment of their consumption [98,99].

### Conclusions

To conclude, seeing the need to increase the effectiveness of conventional approaches, this intervention is the first to use VR as a therapeutic tool in the treatment of CUD in a population with SMD. These preliminary results suggested moderate to high reductions in cannabis use, as well as improved quality of life and psychiatric symptoms over a 1-year period. Indeed, this new avenue is promising since this population’s options are currently very limited. That is even more important for individuals with SMD, for whom cannabis use greatly impacts several spheres of their life (eg, high hospitalization rate, functioning, and quality of life). Although studies have shown that emotional intensity and cravings in VR are similar to those in daily life [100,101], an analysis of the components between the avatars’ dialogue and that of the patient would be necessary to determine whether these components, as well as the dialogical aspect, explain the efficacy of this intervention. Moreover, in order to validate the superiority of this intervention over the existing ones, a larger single-blind randomized controlled trial is underway.

## Supplementary material

10.2196/58499 Checklist 1 CONSORT-EHEALTH checklist (V 1.6.1).

## Abbreviations

B-MACQ: Brief-Marijuana Consequences Questionnaire
CBT: cognitive behavioral therapy
CUD: cannabis use disorder
CUPIT: Cannabis Use Problems Identification Test
*DSM-5*: *Diagnostic and Statistical Manual of Mental Disorders, Fifth Edition*
PANSS: Positive and Negative Syndrome Scale
PBSM-SF: Protective Behavioral Strategies for Marijuana Short Form
QLS: Quality of Life Scale
SMD: severe mental disorder
SUD: substance use disorder
THC: Δ-9-tetrahydrocannabinol
THC-COOH: 11-nor-9-Carboxy-Δ9-tetrahydrocannabinol
TLFB: Timeline Follow-Back
VR: virtual reality
