# Supplementary material for: Avatar Intervention in Virtual Reality for Cannabis Use Disorder in Individuals With Severe Mental Disorders: Results From a 1-Year, Single-Arm Clinical Trial
Source: JMIR Ment Health. 2024 Nov 27;11:e58499. doi: 10.2196/58499 (PMC11612600; doi:10.2196/58499)
Supplement: Checklist 1 [file mental-v11-e58499-s001.pdf]

CONSORT-EHEALTH (V 1.6.1) - Submission/Publication Form

The CONSORT-EHEALTH checklist is intended for authors of randomized trials evaluating web-based and Internet-based applications/interventions, including mobile interventions, electronic games (incl multiplayer games), social media, certain telehealth applications, and other interactive and/or networked electronic applications. Some of the items (e.g. all subitems under item 5 - description of the intervention) may also be applicable for other study designs.

The goal of the CONSORT EHEALTH checklist and guideline is to be
a) a guide for reporting for authors of RCTs,
b) to form a basis for appraisal of an ehealth trial (in terms of validity)

CONSORT-EHEALTH items/subitems are MANDATORY reporting items for studies published in the Journal of Medical Internet Research and other journals / scientific societies endorsing the checklist.

Items numbered 1., 2., 3., 4a., 4b etc are original CONSORT or CONSORT-NPT (non-pharmacologic treatment) items.
Items with Roman numerals (i., ii, iii, iv etc.) are CONSORT-EHEALTH extensions/ clarifications.

As the CONSORT-EHEALTH checklist is still considered in a formative stage, we would ask that you also RATE ON A SCALE OF 1-5 how important/useful you feel each item is FOR THE PURPOSE OF THE CHECKLIST and reporting guideline (optional).

Mandatory reporting items are marked with a red *.
In the textboxes, either copy & paste the relevant sections from your manuscript into this form - please include any quotes from your manuscript in QUOTATION MARKS,
or answer directly by providing additional information not in the manuscript, or elaborating on why the item was not relevant for this study.

YOUR ANSWERS WILL BE PUBLISHED AS A SUPPLEMENTARY FILE TO YOUR PUBLICATION IN JMIR AND ARE CONSIDERED PART OF YOUR PUBLICATION (IF ACCEPTED).
Please fill in these questions diligently. Information will not be copyedited, so please use proper spelling and grammar, use correct capitalization, and avoid abbreviations.

DO NOT FORGET TO SAVE AS PDF _AND_ CLICK THE SUBMIT BUTTON SO YOUR ANSWERS ARE IN OUR DATABASE !!!

Citation Suggestion (if you append the pdf as Appendix we suggest to cite this paper in the caption):

Eysenbach G, CONSORT-EHEALTH Group
CONSORT-EHEALTH: Improving and Standardizing Evaluation Reports of Web-based and Mobile Health Interventions
J Med Internet Res 2011;13(4):e126
URL: http://www.jmir.org/2011/4/e126/
doi: 10.2196/jmir.1923
PMID: 22209829

sabrinagiguere17@gmail.com Changer de compte

Brouillon enregistré

Non partagé

* Indique une question obligatoire

Your name *

First Last

Giguère

Primary Affiliation (short), City, Country *

University of Toronto, Toronto, Canada

University of Montreal, Montreal, Canada

Your e-mail address *

abc@gmail.com

sabrina.giguere.2@umontreal.ca

Title of your manuscript *

Provide the (draft) title of your manuscript.

Avatar Intervention in virtual reality for Cannabis Use Disorder in Individuals withWith Severe Mental Disorders: Results From a One1-Year Single-arm clinical trial

Name of your App/Software/Intervention *

If there is a short and a long/alternate name, write the short name first and add the long name in brackets.

Intervention Avatar for Cannabis Use Disorder

Evaluated Version (if any)

e.g. "V1", "Release 2017-03-01", "Version 2.0.27913"

Votre réponse

Language(s) *

What language is the intervention/app in? If multiple languages are available, separate by comma (e.g. "English, French")

French

URL of your Intervention Website or App

e.g. a direct link to the mobile app on app in appstore (itunes, Google Play), or URL of the website. If the intervention is a DVD or hardware, you can also link to an Amazon page.

https://avatar-intervention.ca/

URL of an image/screenshot (optional)

Votre réponse

Accessibility *

Can an enduser access the intervention presently?

- (selected) access is free and open
- access only for special usergroups, not open
- access is open to everyone, but requires payment/subscription/in-app purchases
- app/intervention no longer accessible
- Autre :

Primary Medical Indication/Disease/Condition *

e.g. "Stress", "Diabetes", or define the target group in brackets after the condition, e.g. "Autism (Parents of children with)", "Alzheimers (Informal Caregivers of)"

cannabis use disorder

Primary Outcomes measured in trial *

comma-separated list of primary outcomes reported in the trial

quantity of cannabis use, frequency of cannab

Secondary/other outcomes

Are there any other outcomes the intervention is expected to affect?

urine levels of 11-nor-9-Carboxy-Δ9-tetrahydrocannabinol, severity of problematic cannabis use, motivation for change cannabis use, strategies employed used to mitigate harms related to cannabis use, negative consequences associated with cannabis, psychiatric symptoms, quality and satisfaction of life, money spent on drugs and alcohol excluding cannabis use

Recommended "Dose" *

What do the instructions for users say on how often the app should be used?

- ( ) Approximately Daily
- (x) Approximately Weekly
- ( ) Approximately Monthly
- ( ) Approximately Yearly
- ( ) "as needed"
- ( ) Autre :

Approx. Percentage of Users (starters) still using the app as recommended after 3 months *

- ○ unknown / not evaluated
- ○ 0-10%
- ○ 11-20%
- ○ 21-30%
- ○ 31-40%
- ○ 41-50%
- ○ 51-60%
- ○ 61-70%
- ○ 71%-80%
- ○ 81-90%
- ○ 91-100%
- ◉ Autre : Participants attended at least 8-weekly sessions intervention

Overall, was the app/intervention effective? *

- ◯ yes: all primary outcomes were significantly better in intervention group vs control
- ◯ partly: SOME primary outcomes were significantly better in intervention group vs control
- ◯ no statistically significant difference between control and intervention
- ◯ potentially harmful: control was significantly better than intervention in one or more outcomes
- ◯ inconclusive: more research is needed
- ◉ Autre : There is no control group

Article Preparation Status/Stage *

At which stage in your article preparation are you currently (at the time you fill in this form)

- ◯ not submitted yet - in early draft status
- ◯ not submitted yet - in late draft status, just before submission
- ◯ submitted to a journal but not reviewed yet
- ◯ submitted to a journal and after receiving initial reviewer comments
- ◉ submitted to a journal and accepted, but not published yet
- ◯ published
- ◯ Autre :

Journal *

If you already know where you will submit this paper (or if it is already submitted), please provide the journal name (if it is not JMIR, provide the journal name under "other")

- ○ not submitted yet / unclear where I will submit this
- ◉ Journal of Medical Internet Research (JMIR)
- ○ JMIR mHealth and UHealth
- ○ JMIR Serious Games
- ○ JMIR Mental Health
- ○ JMIR Public Health
- ○ JMIR Formative Research
- ○ Other JMIR sister journal
- ○ Autre :

Is this a full powered effectiveness trial or a pilot/feasibility trial? *

- ◉ Pilot/feasibility
- ○ Fully powered

Manuscript tracking number *

If this is a JMIR submission, please provide the manuscript tracking number under "other" (The ms tracking number can be found in the submission acknowledgement email, or when you login as author in JMIR. If the paper is already published in JMIR, then the ms tracking number is the four-digit number at the end of the DOI, to be found at the bottom of each published article in JMIR)

- ○ no ms number (yet) / not (yet) submitted to / published in JMIR
- ◉ Autre : 58499

TITLE AND ABSTRACT

1a) TITLE: Identification as a randomized trial in the title

1a) Does your paper address CONSORT item 1a? *

I.e does the title contain the phrase "Randomized Controlled Trial"? (if not, explain the reason under "other")

- ○ yes
- ◉ Autre : This is a single-arm clinical trial with no control group

1a-i) Identify the mode of delivery in the title

Identify the mode of delivery. Preferably use “web-based” and/or “mobile” and/or “electronic game” in the title. Avoid ambiguous terms like “online”, “virtual”, “interactive”. Use “Internet-based” only if Intervention includes non-web-based Internet components (e.g. email), use “computer-based” or “electronic” only if offline products are used. Use “virtual” only in the context of “virtual reality” (3-D worlds). Use “online” only in the context of “online support groups”. Complement or substitute product names with broader terms for the class of products (such as “mobile” or “smart phone” instead of “iphone”), especially if the application runs on different platforms.

	1	2	3	4	5	
subitem not at all important	○	○	○	○	◉	essential

Effacer la sélection

Does your paper address subitem 1a-i? *

Copy and paste relevant sections from manuscript title (include quotes in quotation marks "like this" to indicate direct quotes from your manuscript), or elaborate on this item by providing additional information not in the ms, or briefly explain why the item is not applicable/relevant for your study

Avatar Intervention in "virtual reality" for Cannabis Use Disorder in Individuals With Severe Mental Disorders: Results From a One1-Year Pilot Clinical Trial

1a-ii) Non-web-based components or important co-interventions in title

Mention non-web-based components or important co-interventions in title, if any (e.g., “with telephone support”).

	1	2	3	4	5	
subitem not at all important	◉	○	○	○	○	essential

Effacer la sélection

Does your paper address subitem 1a-ii?

Copy and paste relevant sections from manuscript title (include quotes in quotation marks "like this" to indicate direct quotes from your manuscript), or elaborate on this item by providing additional information not in the ms, or briefly explain why the item is not applicable/relevant for your study

Avatar Intervention in "virtual reality" for Cannabis Use Disorder in Individuals With Severe Mental Disorders: Results From a One1-Year Pilot Clinical Trial

1a-iii) Primary condition or target group in the title

Mention primary condition or target group in the title, if any (e.g., “for children with Type I Diabetes”) Example: A Web-based and Mobile Intervention with Telephone Support for Children with Type I Diabetes: Randomized Controlled Trial

	1	2	3	4	5	
subitem not at all important	○	○	○	○	◉	essential

Effacer la sélection

Does your paper address subitem 1a-iii? *

Copy and paste relevant sections from manuscript title (include quotes in quotation marks "like this" to indicate direct quotes from your manuscript), or elaborate on this item by providing additional information not in the ms, or briefly explain why the item is not applicable/relevant for your study

Avatar Intervention in virtual reality for "Cannabis Use Disorder" in "Individuals With Severe Mental Disorders": Results From a One1-Year Pilot Clinical Trial

1b) ABSTRACT: Structured summary of trial design, methods, results, and conclusions

NPT extension: Description of experimental treatment, comparator, care providers, centers, and blinding status.

1b-i) Key features/functionalities/components of the intervention and comparator in the METHODS section of the ABSTRACT

Mention key features/functionalities/components of the intervention and comparator in the abstract. If possible, also mention theories and principles used for designing the site. Keep in mind the needs of systematic reviewers and indexers by including important synonyms. (Note: Only report in the abstract what the main paper is reporting. If this information is missing from the main body of text, consider adding it)

	1	2	3	4	5	
subitem not at all important	○	○	○	○	◉	essential

Effacer la sélection

Does your paper address subitem 1b-i? *

Copy and paste relevant sections from the manuscript abstract (include quotes in quotation marks "like this" to indicate direct quotes from your manuscript), or elaborate on this item by providing additional information not in the ms, or briefly explain why the item is not applicable/relevant for your study

Over the course of the 8 intervention sessions, "participants were given the opportunity to enter a dialogue in virtual reality with an avatar representing a person with a significant role in their consumption, who was animated in real time by a therapist."

1b-ii) Level of human involvement in the METHODS section of the ABSTRACT

Clarify the level of human involvement in the abstract, e.g., use phrases like “fully automated” vs. “therapist/nurse/care provider/physician-assisted” (mention number and expertise of providers involved, if any). (Note: Only report in the abstract what the main paper is reporting. If this information is missing from the main body of text, consider adding it)

	1	2	3	4	5	
subitem not at all important	○	○	○	○	◉	essential

Effacer la sélection

Does your paper address subitem 1b-ii?

Copy and paste relevant sections from the manuscript abstract (include quotes in quotation marks "like this" to indicate direct quotes from your manuscript), or elaborate on this item by providing additional information not in the ms, or briefly explain why the item is not applicable/relevant for your study

Over the course of the 8 intervention sessions, participants were given the opportunity to enter a dialogue in virtual reality with an avatar representing a person with a significant role in their consumption, "who was animated in real time by a therapist"

1b-iii) Open vs. closed, web-based (self-assessment) vs. face-to-face assessments in the METHODS section of the ABSTRACT

Mention how participants were recruited (online vs. offline), e.g., from an open access website or from a clinic or a closed online user group (closed usergroup trial), and clarify if this was a purely web-based trial, or there were face-to-face components (as part of the intervention or for assessment). Clearly say if outcomes were self-assessed through questionnaires (as common in web-based trials). Note: In traditional offline trials, an open trial (open-label trial) is a type of clinical trial in which both the researchers and participants know which treatment is being administered. To avoid confusion, use “blinded” or “unblinded” to indicated the level of blinding instead of “open”, as “open” in web-based trials usually refers to “open access” (i.e. participants can self-enrol). (Note: Only report in the abstract what the main paper is reporting. If this information is missing from the main body of text, consider adding it)

	1	2	3	4	5	
subitem not at all important	○	◉	○	○	○	essential

Effacer la sélection

Does your paper address subitem 1b-iii?

Copy and paste relevant sections from the manuscript abstract (include quotes in quotation marks "like this" to indicate direct quotes from your manuscript), or elaborate on this item by providing additional information not in the ms, or briefly explain why the item is not applicable/relevant for your study

See method section for futher informations

1b-iv) RESULTS section in abstract must contain use data

Report number of participants enrolled/assessed in each group, the use/uptake of the intervention (e.g., attrition/adherence metrics, use over time, number of logins etc.), in addition to primary/secondary outcomes. (Note: Only report in the abstract what the main paper is reporting. If this information is missing from the main body of text, consider adding it)

	1	2	3	4	5	
subitem not at all important	○	◉	○	○	○	essential

Effacer la sélection

Does your paper address subitem 1b-iv?

Copy and paste relevant sections from the manuscript abstract (include quotes in quotation marks "like this" to indicate direct quotes from your manuscript), or elaborate on this item by providing additional information not in the ms, or briefly explain why the item is not applicable/relevant for your study

See results section for further infomation about the number of participants enrolled/ assessed, the use/uptake of the intervention.

"Significant reductions were observed in the quantity of cannabis consumed, and these were maintained until the 12-month follow-up visit (d =0.804; P<0.001; confirmed by urine quantification). Frequency of cannabis use showed a small significant reduction at the 3-month follow-up (d=0.384; P=0.031). Moreover, improvements were observed for severity of CUD, cannabis -related negative consequences, motivation to change cannabis use, and in the strategies employedused to mitigate harms related to cannabis use. Finally, moderate benefits were observed for quality of life and psychiatric symptoms."

1b-v) CONCLUSIONS/DISCUSSION in abstract for negative trials

Conclusions/Discussions in abstract for negative trials: Discuss the primary outcome - if the trial is negative (primary outcome not changed), and the intervention was not used, discuss whether negative results are attributable to lack of uptake and discuss reasons. (Note: Only report in the abstract what the main paper is reporting. If this information is missing from the main body of text, consider adding it)

	1	2	3	4	5	
subitem not at all important	◉	○	○	○	○	essential

Effacer la sélection

Does your paper address subitem 1b-v?

Copy and paste relevant sections from the manuscript abstract (include quotes in quotation marks "like this" to indicate direct quotes from your manuscript), or elaborate on this item by providing additional information not in the ms, or briefly explain why the item is not applicable/relevant for your study

There are no primary results that have not changed.

INTRODUCTION

2a) In INTRODUCTION: Scientific background and explanation of rationale

2a-i) Problem and the type of system/solution

Describe the problem and the type of system/solution that is object of the study: intended as stand-alone intervention vs. incorporated in broader health care program? Intended for a particular patient population? Goals of the intervention, e.g., being more cost-effective to other interventions, replace or complement other solutions? (Note: Details about the intervention are provided in “Methods” under 5)

	1	2	3	4	5	
subitem not at all important	○	○	○	○	◉	essential

Effacer la sélection

Does your paper address subitem 2a-i? *

Copy and paste relevant sections from the manuscript (include quotes in quotation marks "like this" to indicate direct quotes from your manuscript), or elaborate on this item by providing additional information not in the ms, or briefly explain why the item is not applicable/relevant for your study

"Although cannabis is widely used in the general population, the prevalence of cannabis use is higher among individuals with severe mental disorder [(SMD):; including chronic psychotic and mood disorders. Patients with SMD are more likely to progress to CUD and usually have more difficulty in achieving abstinence. Moreover, this population is more likely to suffer fromendure negative consequences associated with cannabis use, including exacerbation of psychiatric symptoms, reduced adherence to treatment, and increased hospitalization rates [23-29]. Considering that cannabis is being legalized in multiple states in countries and that Δ-9-tétrahydrocannabinol (THC) levels are rising steadily while appearing to be correlated with progression to CUD [30,31], effective treatments are critically needed. Hence, there is an urgent need to develop new treatment modalities adapted to this complex population and whose benefits would be maintained. In recent years, virtual reality (VR) has emerged as a new modality for psychiatric interventions. Indeed, VR interventions allow patients to be immersed gradually in fully personalized and safe environments controlled by the therapist, recreating real situations in which it is possible to interact [51-54] and experience emotions similar to those experienced in life [55,56]. Also, VR overcomes certain limitations of current exposure therapies, where it may not necessarily be safe to expose patients in real life to certain environments and where there may lack professional resources [55]. Finally, VR allows patients to learn and apply strategies in real time, as the emotions generated during the immersion are similar in intensity to those experienced in everyday life, enabling better transposition. To improve the effectiveness of CUD treatments for people with SMD, our research team has developed the avatar intervention for CUD. To the best of our knowledge, this is the first intervention for the treatment of CUD focusing on relational components contributing to substance use while using VR as a personalized therapeutic tool."

2a-ii) Scientific background, rationale: What is known about the (type of) system

Scientific background, rationale: What is known about the (type of) system that is the object of the study (be sure to discuss the use of similar systems for other conditions/ diagnoses, if appropiate), motivation for the study, i.e. what are the reasons for and what is the context for this specific study, from which stakeholder viewpoint is the study performed, potential impact of findings [2]. Briefly justify the choice of the comparator.

	1	2	3	4	5	
subitem not at all important	○	○	○	○	◉	essential

Effacer la sélection

Does your paper address subitem 2a-ii? *

Copy and paste relevant sections from the manuscript (include quotes in quotation marks "like this" to indicate direct quotes from your manuscript), or elaborate on this item by providing additional information not in the ms, or briefly explain why the item is not applicable/relevant for your study

"VR has been used as a therapeutic tool to treat numerous psychiatric symptoms and disorders, such as psychotic disorders, anxiety disorders, obsessive-compulsive disorder, and eating disorders [54,58,59]. Regarding substance use disorders (SUD), the efficacy of such therapeutic options has only been evaluated for nicotine dependence. While simple exposure to the substance in VR was not sufficient to obtain significant results, using VR as an opportunity to learn during immersion showed significant positive effects [60-62]. One crucial limitation common to all currently available VR interventions for SUD remains that that the environments used are not interactive (i.e.,ie, predefined situations and no dialogues in real time with an avatar). The scenarios could consequently not be personalized to the patient's needs. Results from an initial pilot study showed positive short-term benefits of this avatar intervention on cannabis use [63]. Indeed, results showed significant decreases in the quantity of cannabis consumed, as well as in the severity of CUD."

2b) In INTRODUCTION: Specific objectives or hypotheses

Does your paper address CONSORT subitem 2b? *

Copy and paste relevant sections from the manuscript (include quotes in quotation marks "like this" to indicate direct quotes from your manuscript), or elaborate on this item by providing additional information not in the ms, or briefly explain why the item is not applicable/relevant for your study

"The aim of thise present study was to evaluate the effects of the avatar intervention at 3, 6, and 12 months after the end of the intervention."

METHODS

3a) Description of trial design (such as parallel, factorial) including allocation ratio

Does your paper address CONSORT subitem 3a? *

Copy and paste relevant sections from the manuscript (include quotes in quotation marks "like this" to indicate direct quotes from your manuscript), or elaborate on this item by providing additional information not in the ms, or briefly explain why the item is not applicable/relevant for your study

This Single-arm trial study isis part of the clinical trial (ClinicalTrials.gov Identifier:; NCT05726617)

3b) Important changes to methods after trial commencement (such as eligibility criteria), with reasons

Does your paper address CONSORT subitem 3b? *

Copy and paste relevant sections from the manuscript (include quotes in quotation marks "like this" to indicate direct quotes from your manuscript), or elaborate on this item by providing additional information not in the ms, or briefly explain why the item is not applicable/relevant for your study

There was no Important changes to methods after trial commencement

3b-i) Bug fixes, Downtimes, Content Changes

Bug fixes, Downtimes, Content Changes: ehealth systems are often dynamic systems. A description of changes to methods therefore also includes important changes made on the intervention or comparator during the trial (e.g., major bug fixes or changes in the functionality or content) (5-iii) and other “unexpected events” that may have influenced study design such as staff changes, system failures/downtimes, etc. [2].

	1	2	3	4	5	
subitem not at all important	(•)	( )	( )	( )	( )	essential

Effacer la sélection

Does your paper address subitem 3b-i?

Copy and paste relevant sections from the manuscript (include quotes in quotation marks "like this" to indicate direct quotes from your manuscript), or elaborate on this item by providing additional information not in the ms, or briefly explain why the item is not applicable/relevant for your study

There was no changes to methods or important changes made on the intervention during the trial

4a) Eligibility criteria for participants

Does your paper address CONSORT subitem 4a? *

Copy and paste relevant sections from the manuscript (include quotes in quotation marks "like this" to indicate direct quotes from your manuscript), or elaborate on this item by providing additional information not in the ms, or briefly explain why the item is not applicable/relevant for your study

"Participants were eligible if they were 18 years of age or older, had diagnosis of moderate or severe CUD, as well as a diagnosis of a schizophrenia-spectrum disorder (i.e.,ie, schizophrenia, schizoaffective disorder, and unspecified schizophrenia spectrum) or a chronic mood disorder (i.e.,ie, bipolar disorder, and major depressive disorder). The diagnoses were confirmed using the Structured Clinical Iinterview for DSM-5 (SCID-5) [64]. Participants were excluded if they had a neurological disease or an unstable serious physical illness."

4a-i) Computer / Internet literacy

Computer / Internet literacy is often an implicit “de facto” eligibility criterion - this should be explicitly clarified.

	1	2	3	4	5	
subitem not at all important	◉	○	○	○	○	essential

Effacer la sélection

Does your paper address subitem 4a-i?

Copy and paste relevant sections from the manuscript (include quotes in quotation marks "like this" to indicate direct quotes from your manuscript), or elaborate on this item by providing additional information not in the ms, or briefly explain why the item is not applicable/relevant for your study

This is an in-person intervention assisted by a psychiatrist, participants do not need to be proficient in computers or the internet.

4a-ii) Open vs. closed, web-based vs. face-to-face assessments:

Open vs. closed, web-based vs. face-to-face assessments: Mention how participants were recruited (online vs. offline), e.g., from an open access website or from a clinic, and clarify if this was a purely web-based trial, or there were face-to-face components (as part of the intervention or for assessment), i.e., to what degree got the study team to know the participant. In online-only trials, clarify if participants were quasi-anonymous and whether having multiple identities was possible or whether technical or logistical measures (e.g., cookies, email confirmation, phone calls) were used to detect/prevent these.

	1	2	3	4	5	
subitem not at all important	○	○	○	○	◉	essential

Effacer la sélection

Does your paper address subitem 4a-ii? *

Copy and paste relevant sections from the manuscript (include quotes in quotation marks "like this" to indicate direct quotes from your manuscript), or elaborate on this item by providing additional information not in the ms, or briefly explain why the item is not applicable/relevant for your study

"A total of 35 participants (32 outpatients and 3 inpatients) were recruited either from a psychiatric hospital (Institut universitaire en santé mentale de Montréal) or from the community"

"Clinical evaluations were performed in person by a trained psychiatric nurse about one1 week before the beginning of the intervention (baseline), one1 week after the of intervention (post intervention), as well as 3,- - 6,- and 12 months after the end of intervention."

"Participants attended at least 8-weekly sessions (meanmean = =10.2, SDSD = 2.97) lasting between 60 and 90 minutes in person."

4a-iii) Information giving during recruitment

Information given during recruitment. Specify how participants were briefed for recruitment and in the informed consent procedures (e.g., publish the informed consent documentation as appendix, see also item X26), as this information may have an effect on user self-selection, user expectation and may also bias results.

	1	2	3	4	5	
subitem not at all important	○	○	○	○	○	essential

Does your paper address subitem 4a-iii?

Copy and paste relevant sections from the manuscript (include quotes in quotation marks "like this" to indicate direct quotes from your manuscript), or elaborate on this item by providing additional information not in the ms, or briefly explain why the item is not applicable/relevant for your study

"The trial was approved by the institutional ethics committee of the Centre intégré universitaire de santé et de services sociaux de l'Est-de-l'Île-de-Montréal (CEMTL; 2019-1564) and all participants provided informed written consent. Participants received a compensation amount of $20 for each assessment. Data were collected at the Centre intégré universitaire de santé et de services sociaux de l'Est-de-l'Île-de-Montréal and were de-identified."

4b) Settings and locations where the data were collected

Does your paper address CONSORT subitem 4b? *

Copy and paste relevant sections from the manuscript (include quotes in quotation marks "like this" to indicate direct quotes from your manuscript), or elaborate on this item by providing additional information not in the ms, or briefly explain why the item is not applicable/relevant for your study

"Data were collected at the Centre intégré universitaire de santé et de services sociaux de l'Est-de-l'Île-de-Montréal" and were anonymized.

4b-i) Report if outcomes were (self-)assessed through online questionnaires

Clearly report if outcomes were (self-)assessed through online questionnaires (as common in web-based trials) or otherwise.

	1	2	3	4	5	
subitem not at all important	○	○	○	○	◉	essential

Effacer la sélection

Does your paper address subitem 4b-i? *

Copy and paste relevant sections from the manuscript (include quotes in quotation marks "like this" to indicate direct quotes from your manuscript), or elaborate on this item by providing additional information not in the ms, or briefly explain why the item is not applicable/relevant for your study

There were no outcomes that were self-assessed.

"Clinical evaluations were performed in person by a trained psychiatric nurse" about one1 week before the beginning of the intervention (baseline), one1 week after the of intervention (post intervention), as well as 3,- - 6,- and 12 months after the end of intervention.

4b-ii) Report how institutional affiliations are displayed

Report how institutional affiliations are displayed to potential participants [on ehealth media], as affiliations with prestigious hospitals or universities may affect volunteer rates, use, and reactions with regards to an intervention.(Not a required item – describe only if this may bias results)

	1	2	3	4	5	
subitem not at all important	○	○	○	○	○	essential

Does your paper address subitem 4b-ii?

Copy and paste relevant sections from the manuscript (include quotes in quotation marks "like this" to indicate direct quotes from your manuscript), or elaborate on this item by providing additional information not in the ms, or briefly explain why the item is not applicable/relevant for your study

Votre réponse

5) The interventions for each group with sufficient details to allow replication, including how and when they were actually administered

5-i) Mention names, credential, affiliations of the developers, sponsors, and owners

Mention names, credential, affiliations of the developers, sponsors, and owners [6] (if authors/evaluators are owners or developer of the software, this needs to be declared in a “Conflict of interest” section or mentioned elsewhere in the manuscript).

	1	2	3	4	5	
subitem not at all important	○	○	○	○	●	essential

Effacer la sélection

Does your paper address subitem 5-i?

Copy and paste relevant sections from the manuscript (include quotes in quotation marks "like this" to indicate direct quotes from your manuscript), or elaborate on this item by providing additional information not in the ms, or briefly explain why the item is not applicable/relevant for your study

"The intervention including the materials and course of the sessions was described in further details elsewhere [63,65]."

5-ii) Describe the history/development process

Describe the history/development process of the application and previous formative evaluations (e.g., focus groups, usability testing), as these will have an impact on adoption/use rates and help with interpreting results.

	1	2	3	4	5	
subitem not at all important	○	○	○	○	◉	essential

Effacer la sélection

Does your paper address subitem 5-ii?

Copy and paste relevant sections from the manuscript (include quotes in quotation marks "like this" to indicate direct quotes from your manuscript), or elaborate on this item by providing additional information not in the ms, or briefly explain why the item is not applicable/relevant for your study

"This single-arm trial study is part of the clinical trial (ClinicalTrials.gov Identifier:; NCT05726617) [63]. "

5-iii) Revisions and updating

Revisions and updating. Clearly mention the date and/or version number of the application/intervention (and comparator, if applicable) evaluated, or describe whether the intervention underwent major changes during the evaluation process, or whether the development and/or content was “frozen” during the trial. Describe dynamic components such as news feeds or changing content which may have an impact on the replicability of the intervention (for unexpected events see item 3b).

	1	2	3	4	5	
subitem not at all important	○	○	○	○	○	essential

Does your paper address subitem 5-iii?

Copy and paste relevant sections from the manuscript (include quotes in quotation marks "like this" to indicate direct quotes from your manuscript), or elaborate on this item by providing additional information not in the ms, or briefly explain why the item is not applicable/relevant for your study

There were no changes during the evaluation process, or in the development and/or content during the trial.

5-iv) Quality assurance methods

Provide information on quality assurance methods to ensure accuracy and quality of information provided [1], if applicable.

	1	2	3	4	5	
subitem not at all important	○	○	○	◉	○	essential

Effacer la sélection

Does your paper address subitem 5-iv?

Copy and paste relevant sections from the manuscript (include quotes in quotation marks "like this" to indicate direct quotes from your manuscript), or elaborate on this item by providing additional information not in the ms, or briefly explain why the item is not applicable/relevant for your study

"Quantitative analyses were directed and performed by SG, MB, and AD The paper was written by SG, MB, and LD and revised by AD, SP, and KP. All the authors have made substantial contributions. All authors have read and agreed to the published version of the paper."

5-v) Ensure replicability by publishing the source code, and/or providing screenshots/screen-capture video, and/or providing flowcharts of the algorithms used

Ensure replicability by publishing the source code, and/or providing screenshots/screen-capture video, and/or providing flowcharts of the algorithms used. Replicability (i.e., other researchers should in principle be able to replicate the study) is a hallmark of scientific reporting.

	1	2	3	4	5	
subitem not at all important	◉	○	○	○	○	essential

Effacer la sélection

Does your paper address subitem 5-v?

Copy and paste relevant sections from the manuscript (include quotes in quotation marks "like this" to indicate direct quotes from your manuscript), or elaborate on this item by providing additional information not in the ms, or briefly explain why the item is not applicable/relevant for your study

We do not hold the source code. The application was designed by a company.

5-vi) Digital preservation

Digital preservation: Provide the URL of the application, but as the intervention is likely to change or disappear over the course of the years; also make sure the intervention is archived (Internet Archive, webcitation.org, and/or publishing the source code or screenshots/videos alongside the article). As pages behind login screens cannot be archived, consider creating demo pages which are accessible without login.

	1	2	3	4	5	
subitem not at all important	◉	○	○	○	○	essential

Effacer la sélection

Does your paper address subitem 5-vi?

Copy and paste relevant sections from the manuscript (include quotes in quotation marks "like this" to indicate direct quotes from your manuscript), or elaborate on this item by providing additional information not in the ms, or briefly explain why the item is not applicable/relevant for your study

We do not hold the source code. The application was designed by a company.

5-vii) Access

Access: Describe how participants accessed the application, in what setting/context, if they had to pay (or were paid) or not, whether they had to be a member of specific group. If known, describe how participants obtained “access to the platform and Internet” [1]. To ensure access for editors/reviewers/readers, consider to provide a “backdoor” login account or demo mode for reviewers/readers to explore the application (also important for archiving purposes, see vi).

	1	2	3	4	5	
subitem not at all important	○	○	◉	○	○	essential

Effacer la sélection

Does your paper address subitem 5-vii? *

Copy and paste relevant sections from the manuscript (include quotes in quotation marks "like this" to indicate direct quotes from your manuscript), or elaborate on this item by providing additional information not in the ms, or briefly explain why the item is not applicable/relevant for your study

This is a intervention with a psychiatrist in person.

"Participants were eligible if they were 18 years of age or older, had diagnosis of moderate or severe CUD, as well as a DSM-5 diagnosis of a schizophrenia-spectrum disorder (i.e.,ie, schizophrenia, schizoaffective disorder, and unspecified schizophrenia spectrum) and/oror a chronic mood disorder (i.e.,ie, bipolar disorder, and major depressive disorder). The diagnoses were confirmed using the Sstructured Cclinical Iinterview for Diagnostic And Statistical Manual of Mental Disorder 5 th edition (DSM-5) (SCID-5) [64]. Participants were excluded if they had a neurological disease or an unstable serious physical illness. "

5-viii) Mode of delivery, features/functionalities/components of the intervention and comparator, and the theoretical framework

Describe mode of delivery, features/functionalities/components of the intervention and comparator, and the theoretical framework [6] used to design them (instructional strategy [1], behaviour change techniques, persuasive features, etc., see e.g., [7, 8] for terminology). This includes an in-depth description of the content (including where it is coming from and who developed it) [1],” whether [and how] it is tailored to individual circumstances and allows users to track their progress and receive feedback” [6]. This also includes a description of communication delivery channels and – if computer-mediated communication is a component – whether communication was synchronous or asynchronous [6]. It also includes information on presentation strategies [1], including page design principles, average amount of text on pages, presence of hyperlinks to other resources, etc. [1].

	1	2	3	4	5	
subitem not at all important	○	○	○	○	◉	essential

Effacer la sélection

Does your paper address subitem 5-viii? *

Copy and paste relevant sections from the manuscript (include quotes in quotation marks "like this" to indicate direct quotes from your manuscript), or elaborate on this item by providing additional information not in the ms, or briefly explain why the item is not applicable/relevant for your study

"To achieve this, participants create avatars representing significant people associated with their substance use, who will be played by the therapist. The therapist can play the role of a drug dealer, or family member or friend, depending on which avatar the patient wishes to interact with at each session. The therapist who plays the role of each avatar during the sessions used the same vocabulary and expressions as the person represented by the avatar. For example, if the avatar represents a consumer friend, one1 of the strategies was to encourage participants to find alternatives to consumption (e.g.,eg, avatar: We should do something together other than consume, what should we do?). Avatars were created by the participants, with assistance as needed, based on physical characteristics, e.g.,egfor example, facial features, eye, hair and skin color, haircut, etcand so forth. In this way, the personification of avatars and the relational component established between the avatar and the patient allowed to generate personalized cravings and emotions for each participant. "

"They also decided which avatar would be selected as well as the VR environment (in a bar, an apartment, or a park)."

"In immersion, Tthe therapist animated the avatar by having his voice modified in real- time, as well as by controlling the avatar’s facial expression (i.e.,ie, angry, sad, joyful, and fearful)."

5-ix) Describe use parameters

Describe use parameters (e.g., intended “doses” and optimal timing for use). Clarify what instructions or recommendations were given to the user, e.g., regarding timing, frequency, heaviness of use, if any, or was the intervention used ad libitum.

	1	2	3	4	5	
subitem not at all important	○	○	○	○	◉	essential

Effacer la sélection

Does your paper address subitem 5-ix?

Copy and paste relevant sections from the manuscript (include quotes in quotation marks "like this" to indicate direct quotes from your manuscript), or elaborate on this item by providing additional information not in the ms, or briefly explain why the item is not applicable/relevant for your study

"Participants attended at least 8-weekly sessions (meanmean = =10.2, SDSD = 2.97) lasting between 60 and 90 minutes."

5-x) Clarify the level of human involvement

Clarify the level of human involvement (care providers or health professionals, also technical assistance) in the e-intervention or as co-intervention (detail number and expertise of professionals involved, if any, as well as “type of assistance offered, the timing and frequency of the support, how it is initiated, and the medium by which the assistance is delivered”. It may be necessary to distinguish between the level of human involvement required for the trial, and the level of human involvement required for a routine application outside of a RCT setting (discuss under item 21 – generalizability).

	1	2	3	4	5	
subitem not at all important	○	○	○	○	◉	essential

Effacer la sélection

Does your paper address subitem 5-x?

Copy and paste relevant sections from the manuscript (include quotes in quotation marks "like this" to indicate direct quotes from your manuscript), or elaborate on this item by providing additional information not in the ms, or briefly explain why the item is not applicable/relevant for your study

"The intervention was provided by an experienced psychiatrist (AD) with more than 10 years of experience working with this complex population. "

"Preimmersion is the Ssummary of the preceding week and determination of the objective(s) of this intervention session. The participant and the therapist decided face to face together what the aim of the scenario would be."

"The therapist who plays the role of each avatar during the sessions used the same vocabulary and expressions as the person represented by the avatar."

"Postimmersion is the Ddebriefing of the participant’s experience, including the feelings that arose during the immersion."

5-xi) Report any prompts/reminders used

Report any prompts/reminders used: Clarify if there were prompts (letters, emails, phone calls, SMS) to use the application, what triggered them, frequency etc. It may be necessary to distinguish between the level of prompts/reminders required for the trial, and the level of prompts/reminders for a routine application outside of a RCT setting (discuss under item 21 – generalizability).

	1	2	3	4	5	
subitem not at all important	○	◉	○	○	○	essential

Effacer la sélection

Does your paper address subitem 5-xi? *

Copy and paste relevant sections from the manuscript (include quotes in quotation marks "like this" to indicate direct quotes from your manuscript), or elaborate on this item by providing additional information not in the ms, or briefly explain why the item is not applicable/relevant for your study

This is a intervention in person

5-xii) Describe any co-interventions (incl. training/support)

Describe any co-interventions (incl. training/support): Clearly state any interventions that are provided in addition to the targeted eHealth intervention, as ehealth intervention may not be designed as stand-alone intervention. This includes training sessions and support [1]. It may be necessary to distinguish between the level of training required for the trial, and the level of training for a routine application outside of a RCT setting (discuss under item 21 – generalizability.

	1	2	3	4	5	
subitem not at all important	○	○	○	○	○	essential

Does your paper address subitem 5-xii? *

Copy and paste relevant sections from the manuscript (include quotes in quotation marks "like this" to indicate direct quotes from your manuscript), or elaborate on this item by providing additional information not in the ms, or briefly explain why the item is not applicable/relevant for your study

The application does not require external support

6a) Completely defined pre-specified primary and secondary outcome measures, including how and when they were assessed

Does your paper address CONSORT subitem 6a? *

Copy and paste relevant sections from the manuscript (include quotes in quotation marks "like this" to indicate direct quotes from your manuscript), or elaborate on this item by providing additional information not in the ms, or briefly explain why the item is not applicable/relevant for your study

"The primary outcomes were the quantity and the frequency of cannabis consumption, which were both assessed using the Timeline Follow-Back (TLFB). TLFB is a self-reported questionnaire evaluating substance use in the previous week (e.g.,eg, cannabis, alcohol, and amphetamines) and has strong interrater reliability [66,67]. For cannabis use, the number of joints was reported, equivalent to 0.5 gramgram each. Moreover, to confirm the self-report data on the amounts of cannabis consumed by the participants, urine levels of 11-nor-9-Carboxy-Δ9-tetrahydrocannabinol (THC-COOH) were quantified before the intervention, after the intervention, as well as at the 3-months follow-up [68]. The detailed methodology of that test has been described elsewhere [63]. To verify that cannabis had not been replaced by another substance, the consumption of alcohol and other recreational drugs was also monitored. To have a comparable unit of measurement, all quantities were converted to sale prices as reported by participants. Consequently, a non-significant result would indicate that there is no increase in the consumption of other drugs.Secondary outcomes included the severity of problematic cannabis use, motivation for change, protective strategies for cannabis use, consequences of cannabis use, psychiatric symptoms, and quality of life. Problematic cannabis use was assessed using the Cannabis Use Problems Identification Test (CUPIT), a self-reported questionnaire with good to excellent test-retest reliability and internal consistency [69]. The Mmarijuana Lladder was used to measured participants' motivation to change their cannabis use habits. This tool has a good concurrent and predictive validity assessing their readiness to change (pre-contemplation, contemplation, preparation, action, and maintenance) [70]. The strategies employedused to mitigate harms related to cannabis use was measured by Protective Behavioral Strategies for Marijuana Short Form (PBSM-SF), which demonstrated satisfactory fidelity, construct validity, and criterion related validity [71,72]. The negative consequences associated with cannabis use was assessed with the Brief-Marijuana Consequences Questionnaire (B-MACQ) [73]. Quality and satisfaction of life was assessed using the Heinrichs' Quality of Life Scale (QLS), a 21-items tool covering different spheres of quality of life [74]. The Positive And Negative Syndrome Scale (PANSS) was used to evaluated psychiatric symptoms. The PANSS was shown to have a good interrater reliability, an appropriate test-retest reliability, and a high internal consistency [75,76]. Evaluators were trained to administer this scale, and interrated reliabiltyreliability was ensured by conducting consensus ratings on standardized videotapes and real patients. Because a change in pharmacological treatment during the intervention could potentially impact psychiatric symptoms, the participants' medication lists before and after the therapy were compared. As no significant change was observed, this variable was not considered in further analyses."

6a-i) Online questionnaires: describe if they were validated for online use and apply CHERRIES items to describe how the questionnaires were designed/deployed

If outcomes were obtained through online questionnaires, describe if they were validated for online use and apply CHERRIES items to describe how the questionnaires were designed/deployed [9].

	1	2	3	4	5	
subitem not at all important	○	○	○	○	○	essential

Does your paper address subitem 6a-i?

Copy and paste relevant sections from manuscript text

not applicable; "Clinical evaluations were performed in person by a trained psychiatric nurse"

6a-ii) Describe whether and how “use” (including intensity of use/dosage) was defined/measured/monitored

Describe whether and how “use” (including intensity of use/dosage) was defined/measured/monitored (logins, logfile analysis, etc.). Use/adoption metrics are important process outcomes that should be reported in any ehealth trial.

	1	2	3	4	5	
subitem not at all important	○	○	●	○	○	essential

Effacer la sélection

Does your paper address subitem 6a-ii?

Copy and paste relevant sections from manuscript text

This is a intervention in person

6a-iii) Describe whether, how, and when qualitative feedback from participants was obtained

Describe whether, how, and when qualitative feedback from participants was obtained (e.g., through emails, feedback forms, interviews, focus groups).

	1	2	3	4	5	
subitem not at all important	○	○	○	○	◉	essential

Effacer la sélection

Does your paper address subitem 6a-iii?

Copy and paste relevant sections from manuscript text

"Clinical evaluations were performed in person by a trained psychiatric nurse about one1 week before the beginning of the intervention (baseline), one1 week after the of intervention (post intervention), as well as 3,- - 6,- and 12 months after the end of intervention. To comply with the timeframes for the follow-up period, assessments had to be carried out within a maximum of one1 month to be included."

6b) Any changes to trial outcomes after the trial commenced, with reasons

Does your paper address CONSORT subitem 6b? *

Copy and paste relevant sections from the manuscript (include quotes in quotation marks "like this" to indicate direct quotes from your manuscript), or elaborate on this item by providing additional information not in the ms, or briefly explain why the item is not applicable/relevant for your study

There were not changes to trial outcomes after the trial commenced

7a) How sample size was determined

NPT: When applicable, details of whether and how the clustering by care provides or centers was addressed

7a-i) Describe whether and how expected attrition was taken into account when calculating the sample size

Describe whether and how expected attrition was taken into account when calculating the sample size.

	1	2	3	4	5	
subitem not at all important	○	○	●	○	○	essential

Effacer la sélection

Does your paper address subitem 7a-i?

Copy and paste relevant sections from manuscript title (include quotes in quotation marks "like this" to indicate direct quotes from your manuscript), or elaborate on this item by providing additional information not in the ms, or briefly explain why the item is not applicable/relevant for your study

This is a single-arm clinical trial, the simple size was not previously calculated.

7b) When applicable, explanation of any interim analyses and stopping guidelines

Does your paper address CONSORT subitem 7b? *

Copy and paste relevant sections from the manuscript (include quotes in quotation marks "like this" to indicate direct quotes from your manuscript), or elaborate on this item by providing additional information not in the ms, or briefly explain why the item is not applicable/relevant for your study

There were not interim analyses and stopping guidelines

8a) Method used to generate the random allocation sequence

NPT: When applicable, how care providers were allocated to each trial group

Does your paper address CONSORT subitem 8a? *

Copy and paste relevant sections from the manuscript (include quotes in quotation marks "like this" to indicate direct quotes from your manuscript), or elaborate on this item by providing additional information not in the ms, or briefly explain why the item is not applicable/relevant for your study

There was no control group

8b) Type of randomisation; details of any restriction (such as blocking and block size)

Does your paper address CONSORT subitem 8b? *

Copy and paste relevant sections from the manuscript (include quotes in quotation marks "like this" to indicate direct quotes from your manuscript), or elaborate on this item by providing additional information not in the ms, or briefly explain why the item is not applicable/relevant for your study

There was no control group

9) Mechanism used to implement the random allocation sequence (such as sequentially numbered containers), describing any steps taken to conceal the sequence until interventions were assigned

Does your paper address CONSORT subitem 9? *

Copy and paste relevant sections from the manuscript (include quotes in quotation marks "like this" to indicate direct quotes from your manuscript), or elaborate on this item by providing additional information not in the ms, or briefly explain why the item is not applicable/relevant for your study

There was no control group

10) Who generated the random allocation sequence, who enrolled participants, and who assigned participants to interventions

Does your paper address CONSORT subitem 10? *

Copy and paste relevant sections from the manuscript (include quotes in quotation marks "like this" to indicate direct quotes from your manuscript), or elaborate on this item by providing additional information not in the ms, or briefly explain why the item is not applicable/relevant for your study

There was no control group

11a) If done, who was blinded after assignment to interventions (for example, participants, care providers, those assessing outcomes) and how

NPT: Whether or not administering co-interventions were blinded to group assignment

11a-i) Specify who was blinded, and who wasn't

Specify who was blinded, and who wasn't. Usually, in web-based trials it is not possible to blind the participants [1, 3] (this should be clearly acknowledged), but it may be possible to blind outcome assessors, those doing data analysis or those administering co-interventions (if any).

	1	2	3	4	5	
subitem not at all important	○	○	○	◉	○	essential

Effacer la sélection

Does your paper address subitem 11a-i? *

Copy and paste relevant sections from the manuscript (include quotes in quotation marks "like this" to indicate direct quotes from your manuscript), or elaborate on this item by providing additional information not in the ms, or briefly explain why the item is not applicable/relevant for your study

There was no control group

11a-ii) Discuss e.g., whether participants knew which intervention was the "intervention of interest" and which one was the "comparator"

Informed consent procedures (4a-ii) can create biases and certain expectations - discuss e.g., whether participants knew which intervention was the "intervention of interest" and which one was the "comparator".

	1	2	3	4	5	
subitem not at all important	○	○	○	◉	○	essential

Effacer la sélection

Does your paper address subitem 11a-ii?

Copy and paste relevant sections from the manuscript (include quotes in quotation marks "like this" to indicate direct quotes from your manuscript), or elaborate on this item by providing additional information not in the ms, or briefly explain why the item is not applicable/relevant for your study

There was no control group

11b) If relevant, description of the similarity of interventions

(this item is usually not relevant for ehealth trials as it refers to similarity of a placebo or sham intervention to a active medication/intervention)

Does your paper address CONSORT subitem 11b? *

Copy and paste relevant sections from the manuscript (include quotes in quotation marks "like this" to indicate direct quotes from your manuscript), or elaborate on this item by providing additional information not in the ms, or briefly explain why the item is not applicable/relevant for your study

There was no control group

12a) Statistical methods used to compare groups for primary and secondary outcomes

NPT: When applicable, details of whether and how the clustering by care providers or centers was addressed

Does your paper address CONSORT subitem 12a? *

Copy and paste relevant sections from the manuscript (include quotes in quotation marks "like this" to indicate direct quotes from your manuscript), or elaborate on this item by providing additional information not in the ms, or briefly explain why the item is not applicable/relevant for your study

" Statistical analyses were performed with IBM's Statistical Program for Social Sciences (SPSS) (Vversion 28,; IBM Corp, Armonk, NY, United States). Changes in reported outcomes during the assessment periods before the intervention, post intervention, and 3-, 6, and 12 months after the end of the intervention were assessed using a linear mixed-effects model with maximum- likelihood estimations for missing data [77-79]. Of note, it was expected that the post intervention results might slightly differ from previously published results as follow-up timepoints were also considered in the statistical model [63]. Quantification of THC-COOH in urine was performed by applying the creatinine concentration ratio. Changes in THC-COOH were assessed using Wilcoxon for paired variables. To assess whether participants who dropped out before the end of the protocol (12-month follow-up) had a particular profile, baseline sociodemographic, and clinical characteristics were analyzed. For continuous variables, a t- test was performed for normally distributed data and a Mann–-Whitney test for non-normally distributed data. For dichotomous variables, a Pearson's chi-squared test was used to assess categorical outcomes differences. In thise case, one1 cell or more in the contingency table had a frequency under 5, Fisher's exact test was used as it is more applicable to smaller samples [80]. Although the intervention was planned for 8 sessions, keeping in mind that this was a pilot project, the therapist remained flexible and adapted the number of sessions to each participant (range: 8–-15). The association between the number of sessions and efficacy of primary outcomes (quantity and frequency of cannabis use) was, therefore, examined. To do so, Spearman correlations were calculated between the number of sessions and primary outcomes (i.e.ie, the variation in quantity and frequency of cannabis use). The following descriptions of the strength of reported Cohen's d were used: —small (0.2--0.5), medium (0.5--0.8), and large (≥0.8) [81] and the statistical threshold for significance was set at pP<0.05."

12a-i) Imputation techniques to deal with attrition / missing values

Imputation techniques to deal with attrition / missing values: Not all participants will use the intervention/comparator as intended and attrition is typically high in ehealth trials. Specify how participants who did not use the application or dropped out from the trial were treated in the statistical analysis (a complete case analysis is strongly discouraged, and simple imputation techniques such as LOCF may also be problematic [4]).

	1	2	3	4	5	
subitem not at all important	○	○	○	●	○	essential

Effacer la sélection

Does your paper address subitem 12a-i? *

Copy and paste relevant sections from the manuscript (include quotes in quotation marks "like this" to indicate direct quotes from your manuscript), or elaborate on this item by providing additional information not in the ms, or briefly explain why the item is not applicable/relevant for your study

"Changes in reported outcomes during the assessment periods before the intervention, post intervention, and 3-, 6, and 12 months after the end of the intervention were assessed using a linear mixed-effects model with maximum- likelihood estimations for missing data [77-79].
"

12b) Methods for additional analyses, such as subgroup analyses and adjusted analyses

Does your paper address CONSORT subitem 12b? *

Copy and paste relevant sections from the manuscript (include quotes in quotation marks "like this" to indicate direct quotes from your manuscript), or elaborate on this item by providing additional information not in the ms, or briefly explain why the item is not applicable/relevant for your study

"Concerning the profile of participants who completed the 12-month follow-up (n=13) compared to non-completers (n=19) analyses showed no statistically significant socio-demographic or clinical. At baseline, the amount and frequency of cannabis use, severity of CUD, and motivation to change cannabis use were not significant. However, an amphetamine use disorder was significantly associated with quitting (pP = =0.038), while alcohol and cocaine use disorders were not. Finally, the number of sessions was not significantly correlated with the two2 main outcomes (reduction in quantity and frequency of cannabis use) at each follow-up when compared to baseline data."

X26) REB/IRB Approval and Ethical Considerations [recommended as subheading under "Methods"] (not a CONSORT item)

X26-i) Comment on ethics committee approval

	1	2	3	4	5	
subitem not at all important	○	○	○	○	◉	essential

Effacer la sélection

Does your paper address subitem X26-i?

Copy and paste relevant sections from the manuscript (include quotes in quotation marks "like this" to indicate direct quotes from your manuscript), or elaborate on this item by providing additional information not in the ms, or briefly explain why the item is not applicable/relevant for your study

"The trial was approved by the institutional ethics committee of the Centre intégré universitaire de santé et de services sociaux de l'Est-de-l'Île-de-Montréal (CEMTL; 2019-1564)"

x26-ii) Outline informed consent procedures

Outline informed consent procedures e.g., if consent was obtained offline or online (how? Checkbox, etc.?), and what information was provided (see 4a-ii). See [6] for some items to be included in informed consent documents.

	1	2	3	4	5	
subitem not at all important	○	◉	○	○	○	essential

Effacer la sélection

Does your paper address subitem X26-ii?

Copy and paste relevant sections from the manuscript (include quotes in quotation marks "like this" to indicate direct quotes from your manuscript), or elaborate on this item by providing additional information not in the ms, or briefly explain why the item is not applicable/relevant for your study

"all participants provided informed written consent in person"

X26-iii) Safety and security procedures

Safety and security procedures, incl. privacy considerations, and any steps taken to reduce the likelihood or detection of harm (e.g., education and training, availability of a hotline)

	1	2	3	4	5	
subitem not at all important	○	◉	○	○	○	essential

Effacer la sélection

Does your paper address subitem X26-iii?

Copy and paste relevant sections from the manuscript (include quotes in quotation marks "like this" to indicate direct quotes from your manuscript), or elaborate on this item by providing additional information not in the ms, or briefly explain why the item is not applicable/relevant for your study

"Data were collected at the Centre intégré universitaire de santé et de services sociaux de l'Est-de-l'Île-de-Montréal and were anonymized. "

RESULTS

13a) For each group, the numbers of participants who were randomly assigned, received intended treatment, and were analysed for the primary outcome

NPT: The number of care providers or centers performing the intervention in each group and the number of patients treated by each care provider in each center

Does your paper address CONSORT subitem 13a? *

Copy and paste relevant sections from the manuscript (include quotes in quotation marks "like this" to indicate direct quotes from your manuscript), or elaborate on this item by providing additional information not in the ms, or briefly explain why the item is not applicable/relevant for your study

"The intervention was provided by an experienced psychiatrist (AD) with more than 10 years of experience working with this complex population."
"A total of 35 participants (32 outpatients and 3 inpatients) were recruited either from a psychiatric hospital (Institut universitaire en santé mentale de Montréal) or from the community (Figure 1)."

"Detailed sample characteristics can be found in Table 1."

13b) For each group, losses and exclusions after randomisation, together with reasons

Does your paper address CONSORT subitem 13b? (NOTE: Preferably, this is shown in a CONSORT flow diagram) *

Copy and paste relevant sections from the manuscript (include quotes in quotation marks "like this" to indicate direct quotes from your manuscript), or elaborate on this item by providing additional information not in the ms, or briefly explain why the item is not applicable/relevant for your study

"See figure 1"

Your answer must have a minimum of 25 characters.

13b-i) Attrition diagram

Strongly recommended: An attrition diagram (e.g., proportion of participants still logging in or using the intervention/comparator in each group plotted over time, similar to a survival curve) or other figures or tables demonstrating usage/dose/engagement.

	1	2	3	4	5	
subitem not at all important	○	○	○	○	◉	essential

Effacer la sélection

Does your paper address subitem 13b-i?

Copy and paste relevant sections from the manuscript or cite the figure number if applicable (include quotes in quotation marks "like this" to indicate direct quotes from your manuscript), or elaborate on this item by providing additional information not in the ms, or briefly explain why the item is not applicable/relevant for your study

"See figure 1"

Your answer must have a minimum of 25 characters.

14a) Dates defining the periods of recruitment and follow-up

Does your paper address CONSORT subitem 14a? *

Copy and paste relevant sections from the manuscript (include quotes in quotation marks "like this" to indicate direct quotes from your manuscript), or elaborate on this item by providing additional information not in the ms, or briefly explain why the item is not applicable/relevant for your study

"The study took place from October 2019 to May 2023."

14a-i) Indicate if critical “secular events” fell into the study period

Indicate if critical “secular events” fell into the study period, e.g., significant changes in Internet resources available or “changes in computer hardware or Internet delivery resources”

	1	2	3	4	5	
subitem not at all important	○	○	○	○	◉	essential

Effacer la sélection

Does your paper address subitem 14a-i?

Copy and paste relevant sections from the manuscript (include quotes in quotation marks "like this" to indicate direct quotes from your manuscript), or elaborate on this item by providing additional information not in the ms, or briefly explain why the item is not applicable/relevant for your study

There were no critical “secular events” fell into the study period

14b) Why the trial ended or was stopped (early)

Does your paper address CONSORT subitem 14b? *

Copy and paste relevant sections from the manuscript (include quotes in quotation marks "like this" to indicate direct quotes from your manuscript), or elaborate on this item by providing additional information not in the ms, or briefly explain why the item is not applicable/relevant for your study

The trial was not ended or was stopped (early)

15) A table showing baseline demographic and clinical characteristics for each group
NPT: When applicable, a description of care providers (case volume, qualification, expertise, etc.) and centers (volume) in each group

Does your paper address CONSORT subitem 15? *

Copy and paste relevant sections from the manuscript (include quotes in quotation marks "like this" to indicate direct quotes from your manuscript), or elaborate on this item by providing additional information not in the ms, or briefly explain why the item is not applicable/relevant for your study

"The intervention was provided by an experienced psychiatrist (AD) with more than 10 years of experience working with this complex population. "

"Detailed sample characteristics can be found in Table 1. Participants were mostly men, Caucasian, and unemployed. Most participants had a primary diagnosis of a schizophrenia spectrum disorder. Moreover, most of the subsample had an antisocial or borderline personality disorder. The age ranged between 23 toand 58 years old. The average age of cannabis initiation was 14.7 years old. Almost all participants had a severe CUD. A significant proportion of participants had another substance use disorderSUD, notably alcohol and stimulants (Table 1)."

15-i) Report demographics associated with digital divide issues

In ehealth trials it is particularly important to report demographics associated with digital divide issues, such as age, education, gender, social-economic status, computer/Internet/ehealth literacy of the participants, if known.

	1	2	3	4	5	
subitem not at all important	○	○	○	○	○	essential

Does your paper address subitem 15-i? *

Copy and paste relevant sections from the manuscript (include quotes in quotation marks "like this" to indicate direct quotes from your manuscript), or elaborate on this item by providing additional information not in the ms, or briefly explain why the item is not applicable/relevant for your study

This is a intervention in person. Participants do not need to have knowledge of technology to participate in the intervention.

16) For each group, number of participants (denominator) included in each analysis and whether the analysis was by original assigned groups

16-i) Report multiple “denominators” and provide definitions

Report multiple “denominators” and provide definitions: Report N’s (and effect sizes) “across a range of study participation [and use] thresholds” [1], e.g., N exposed, N consented, N used more than x times, N used more than y weeks, N participants “used” the intervention/comparator at specific pre-defined time points of interest (in absolute and relative numbers per group). Always clearly define “use” of the intervention.

	1	2	3	4	5	
subitem not at all important	○	○	○	●	○	essential

Effacer la sélection

Does your paper address subitem 16-i? *

Copy and paste relevant sections from the manuscript (include quotes in quotation marks "like this" to indicate direct quotes from your manuscript), or elaborate on this item by providing additional information not in the ms, or briefly explain why the item is not applicable/relevant for your study

"Participants attended at least 8-weekly sessions (meanmean = =10.2, SDSD = 2.97) lasting between 60 and 90 minutes."

Detailed sample characteristics can be found in Table 1.

16-ii) Primary analysis should be intent-to-treat

Primary analysis should be intent-to-treat, secondary analyses could include comparing only "users", with the appropriate caveats that this is no longer a randomized sample (see 18-i).

	1	2	3	4	5	
subitem not at all important	○	○	○	○	○	essential

Does your paper address subitem 16-ii?

Copy and paste relevant sections from the manuscript (include quotes in quotation marks "like this" to indicate direct quotes from your manuscript), or elaborate on this item by providing additional information not in the ms, or briefly explain why the item is not applicable/relevant for your study

This is a single-arm clinical trial.

17a) For each primary and secondary outcome, results for each group, and the estimated effect size and its precision (such as 95% confidence interval)

Does your paper address CONSORT subitem 17a? *

Copy and paste relevant sections from the manuscript (include quotes in quotation marks "like this" to indicate direct quotes from your manuscript), or elaborate on this item by providing additional information not in the ms, or briefly explain why the item is not applicable/relevant for your study

"The avatar intervention showed numerous benefits that persisted over time (Table 2). Indeed, a significant reduction in the quantity of cannabis consumed (joints) was observed at all timepoints. Indeed, improvements continued to be seen over time, and the largest effect size was observed at the 12-month follow-up visit (d=0.804,; pP<0.001). The urinary THC-COOH quantification confirmed this reduction in 13 participants from whom urine samples were taken at baseline (meanmean = 708.85, SDSD = 1099.91), post intervention (meanmean = 455.83, SDSD = 622.47,; pP=0.0374), and 3-months follow-up (meanmean = 252.61, SDSD = 343.49,; pP=0.0192). Additionally, there was a strong correlation between THC-COOH quantification and self-reported amounts of cannabis (r=0.791;, pP<0.001). A significant reduction in the frequency of cannabis use was observed at 3-month follow-up compared to baseline (d=0.384,; pP=0.031); however, these improvements were no longer seen during the subsequent follow-ups. Cannabis use was not replaced by another substance as the amount of money spent on all other substances combined (alcohol and drugs, excluding cannabis) decreased from CAD 62.00 to CAD 13.2 at 12-month follow-up. Regarding the severity of problematic cannabis use, a reduction corresponding to a moderate effect size was obtained at all time-points. A moderate increase in motivation to change in cannabis use at post intervention and at 3-month follow-up were observed but this increase motivation has not remained significant at 6 and 12 months. Also, a significant improvement in the strategies employedused to mitigate harms related to cannabis use was observed at all times points. Indeed, improvements continued to be seen over time, and the largest effect size was observed at the 12-month follow-up visit (d=0.769,; pP<0.001). A reduction in negative consequences associated with cannabis compared to the baseline was observed which is significant from the 6-month follow-up. Participants experienced important improvement in quality of life which is significant at 3- and 12 months (d=0.675,; pP=0.004; and d=0.532,; pP=0.0349) and a non-significant trend in post intervention and 6-months. From 3-month follow-up, moderate improvement in psychiatric symptoms were observed on psychiatric symptoms and continued to be seen over time. In particular, there was a significant reduction in positive and negative symptoms at 3-month follow-up, as well as a reduction in general symptoms at all follow-ups. "

17a-i) Presentation of process outcomes such as metrics of use and intensity of use

In addition to primary/secondary (clinical) outcomes, the presentation of process outcomes such as metrics of use and intensity of use (dose, exposure) and their operational definitions is critical. This does not only refer to metrics of attrition (13-b) (often a binary variable), but also to more continuous exposure metrics such as “average session length”. These must be accompanied by a technical description how a metric like a “session” is defined (e.g., timeout after idle time) [1] (report under item 6a).

	1	2	3	4	5	
subitem not at all important	○	○	○	○	○	essential

Does your paper address subitem 17a-i?

Copy and paste relevant sections from the manuscript (include quotes in quotation marks "like this" to indicate direct quotes from your manuscript), or elaborate on this item by providing additional information not in the ms, or briefly explain why the item is not applicable/relevant for your study

"Although the intervention was planned for 8 sessions, keeping in mind that this was a pilot project, the therapist remained flexible and adapted the number of sessions to each participant (range: 8–-15). The association between the number of sessions and efficacy of primary outcomes (quantity and frequency of cannabis use) was, therefore, examined. To do so, Spearman correlations were calculated between the number of sessions and primary outcomes (i.e.ie, the variation in quantity and frequency of cannabis use)."

"Finally, the number of sessions was not significantly correlated with the two2 main outcomes (reduction in quantity and frequency of cannabis use) at each follow-up when compared to baseline data."

17b) For binary outcomes, presentation of both absolute and relative effect sizes is recommended

Does your paper address CONSORT subitem 17b? *

Copy and paste relevant sections from the manuscript (include quotes in quotation marks "like this" to indicate direct quotes from your manuscript), or elaborate on this item by providing additional information not in the ms, or briefly explain why the item is not applicable/relevant for your study

mean, standard deviation, p-value and effect size were presented in table 2.

18) Results of any other analyses performed, including subgroup analyses and adjusted analyses, distinguishing pre-specified from exploratory

Does your paper address CONSORT subitem 18? *

Copy and paste relevant sections from the manuscript (include quotes in quotation marks "like this" to indicate direct quotes from your manuscript), or elaborate on this item by providing additional information not in the ms, or briefly explain why the item is not applicable/relevant for your study

"Concerning the profile of participants who completed the 12-month follow-up (n=13) compared to non-completers (n=19) analyses showed no statistically significant socio-demographic or clinical baseline profil. At baseline, the amount and frequency of cannabis use, severity of CUD, and motivation to change cannabis use were not significant. However, an amphetamine use disorder was significantly associated with quitting (pP = =0.038), while alcohol and cocaine use disorders were not. Finally, the number of sessions was not significantly correlated with the two2 main outcomes (reduction in quantity and frequency of cannabis use) at each follow-up when compared to baseline data."

18-i) Subgroup analysis of comparing only users

A subgroup analysis of comparing only users is not uncommon in ehealth trials, but if done, it must be stressed that this is a self-selected sample and no longer an unbiased sample from a randomized trial (see 16-iii).

	1	2	3	4	5	
subitem not at all important	○	○	○	○	○	essential

Does your paper address subitem 18-i?

Copy and paste relevant sections from the manuscript (include quotes in quotation marks "like this" to indicate direct quotes from your manuscript), or elaborate on this item by providing additional information not in the ms, or briefly explain why the item is not applicable/relevant for your study

this is a single-arm clinical trial

19) All important harms or unintended effects in each group
(for specific guidance see CONSORT for harms)

Does your paper address CONSORT subitem 19? *

Copy and paste relevant sections from the manuscript (include quotes in quotation marks "like this" to indicate direct quotes from your manuscript), or elaborate on this item by providing additional information not in the ms, or briefly explain why the item is not applicable/relevant for your study

"Finally, adverse events were systematically recorded, showing that none of the participants who discontinued [63]."

19-i) Include privacy breaches, technical problems

Include privacy breaches, technical problems. This does not only include physical “harm” to participants, but also incidents such as perceived or real privacy breaches [1], technical problems, and other unexpected/unintended incidents. “Unintended effects” also includes unintended positive effects [2].

	1	2	3	4	5	
subitem not at all important	○	○	○	○	○	essential

Does your paper address subitem 19-i?

Copy and paste relevant sections from the manuscript (include quotes in quotation marks "like this" to indicate direct quotes from your manuscript), or elaborate on this item by providing additional information not in the ms, or briefly explain why the item is not applicable/relevant for your study

There are no privacy breaches and technical problems during the trial

19-ii) Include qualitative feedback from participants or observations from staff/ researchers

Include qualitative feedback from participants or observations from staff/researchers, if available, on strengths and shortcomings of the application, especially if they point to unintended/unexpected effects or uses. This includes (if available) reasons for why people did or did not use the application as intended by the developers.

	1	2	3	4	5	
subitem not at all important	○	○	○	○	○	essential

Does your paper address subitem 19-ii?

Copy and paste relevant sections from the manuscript (include quotes in quotation marks "like this" to indicate direct quotes from your manuscript), or elaborate on this item by providing additional information not in the ms, or briefly explain why the item is not applicable/relevant for your study

"semi-structured interviews were conducted until data saturation (n=11) to evaluate the participants" satisfaction with each component of the intervention (e.g.eg content, frequency, number and sequence of sessions, appropriateness, and use of VR). Overall, the level of satisfaction was found to be high."

DISCUSSION

22) Interpretation consistent with results, balancing benefits and harms, and considering other relevant evidence

NPT: In addition, take into account the choice of the comparator, lack of or partial blinding, and unequal expertise of care providers or centers in each group

22-i) Restate study questions and summarize the answers suggested by the data, starting with primary outcomes and process outcomes (use)

Restate study questions and summarize the answers suggested by the data, starting with primary outcomes and process outcomes (use).

	1	2	3	4	5	
subitem not at all important	○	○	○	○	○	essential

Does your paper address subitem 22-i? *

Copy and paste relevant sections from the manuscript (include quotes in quotation marks "like this" to indicate direct quotes from your manuscript), or elaborate on this item by providing additional information not in the ms, or briefly explain why the item is not applicable/relevant for your study

"In the context in which there is a need for effective interventions with long-term benefits for CUD in a population with SMD, thise present articlestudy aimed to evaluate the effects of a novel VR intervention to treat CUD for up to one year following the end of the intervention. Although avatar intervention for CUD links several pre-existing concepts (e.g.,eg, motivational approach, cognitive-behavioral approach, and relapse prevention), its particularity is that it allows patients to practice their strategies in a safe environment in which cravings and emotions are elicited in a similar intensity to that of their daily life. This intervention’s relational and immersive aspects, which are entirely personalized, are hypothesized to enable the transposition of learnings into everyday life [57,58], leading to significant improvements. Indeed, results from thise present study show significant reductions in the total amount and frequency of cannabis use, which were maintained over time. Improvements were also noted in the severity of problematic cannabis use, negative consequences, motivation for change, psychotic symptoms, and quality of life. "

22-ii) Highlight unanswered new questions, suggest future research

Highlight unanswered new questions, suggest future research.

	1	2	3	4	5	
subitem not at all important	◯	◯	◯	◯	◯	essential

Does your paper address subitem 22-ii?

Copy and paste relevant sections from the manuscript (include quotes in quotation marks "like this" to indicate direct quotes from your manuscript), or elaborate on this item by providing additional information not in the ms, or briefly explain why the item is not applicable/relevant for your study

"As the specific mechanisms of this new intervention have yet to be elucidated in comparison with a conventional face-to-face intervention, these will need to be more deeply investigated in future studies."

"Therefore, a cost-effectiveness analysis of VR use for CUD treatment in a population with SMD will be necessary prior to implementing this intervention in regular clinical practice [31,92,93]."

20) Trial limitations, addressing sources of potential bias, imprecision, and, if relevant, multiplicity of analyses

20-i) Typical limitations in ehealth trials

Typical limitations in ehealth trials: Participants in ehealth trials are rarely blinded. Ehealth trials often look at a multiplicity of outcomes, increasing risk for a Type I error. Discuss biases due to non-use of the intervention/usability issues, biases through informed consent procedures, unexpected events.

	1	2	3	4	5	
subitem not at all important	◯	◯	◯	◯	◯	essential

Does your paper address subitem 20-i? *

Copy and paste relevant sections from the manuscript (include quotes in quotation marks "like this" to indicate direct quotes from your manuscript), or elaborate on this item by providing additional information not in the ms, or briefly explain why the item is not applicable/relevant for your study

"While the results suggest that the avatar intervention for CUD appears to be a promising avenue in individuals with SMD and CUD, the present trial has some important limitations to highlight. Firstly, this study does not include a control group for comparison purposes. Considering studies have observed a regression towards the mean of psychiatric symptoms, naturally, in assessments with several timepoints as well as to control the non-specific effects of psychotherapies, a control group intervention will be necessary in future clinical trials [96,97]. Secondly, as there was no control group, the evaluators could not be blind to the intervention. Thirdly, as this was a pilot trial, the sample size was small. To address these three major limitations, a single-blind randomized controlled trial with a projected enrolment of 150 participants comparing the avatar intervention for CUD with a conventional addiction intervention is underway. Fourthly, the THC/CBD ratio was not considered, mainly because participants generally did n"ot know exactly what they were consuming. A potential solution to this problem would be to analyze a sample of each participant's cannabis; however, this raises ethical and legal issues that could be complex to resolve. Fifthly, in this first clinical pilot, the interventions were offered by a single therapist. In the future, it is planned to integrate other service providers and to harmonize the intervention by means of training and an intervention manual. Finally, in the interests of desirability, patients may have underestimated their consumption. However, quantitative analyses of THC-COOH undertaken at baseline, post and 3-month follow-up show a strong correlation with patients" self-reported measures (as observed in other studies using similar quantification methods), indicating that they have a good assessment of their consumption [98,99]."

21) Generalisability (external validity, applicability) of the trial findings

NPT: External validity of the trial findings according to the intervention, comparators, patients, and care providers or centers involved in the trial

21-i) Generalizability to other populations

Generalizability to other populations: In particular, discuss generalizability to a general Internet population, outside of a RCT setting, and general patient population, including applicability of the study results for other organizations

	1	2	3	4	5	
subitem not at all important	○	○	○	○	○	essential

Does your paper address subitem 21-i?

Copy and paste relevant sections from the manuscript (include quotes in quotation marks "like this" to indicate direct quotes from your manuscript), or elaborate on this item by providing additional information not in the ms, or briefly explain why the item is not applicable/relevant for your study

Votre réponse

21-ii) Discuss if there were elements in the RCT that would be different in a routine application setting

Discuss if there were elements in the RCT that would be different in a routine application setting (e.g., prompts/reminders, more human involvement, training sessions or other co-interventions) and what impact the omission of these elements could have on use, adoption, or outcomes if the intervention is applied outside of a RCT setting.

	1	2	3	4	5	
subitem not at all important	○	○	○	○	○	essential

Does your paper address subitem 21-ii?

Copy and paste relevant sections from the manuscript (include quotes in quotation marks "like this" to indicate direct quotes from your manuscript), or elaborate on this item by providing additional information not in the ms, or briefly explain why the item is not applicable/relevant for your study

Votre réponse

OTHER INFORMATION

23) Registration number and name of trial registry

Does your paper address CONSORT subitem 23? *

Copy and paste relevant sections from the manuscript (include quotes in quotation marks "like this" to indicate direct quotes from your manuscript), or elaborate on this item by providing additional information not in the ms, or briefly explain why the item is not applicable/relevant for your study

linicalTrials.gov ID NCT05726617, Avatar Intervention for the Treatment of Cannabis Use Disorder in Patients With Severe Mental Health Disorders (Avatar-CUD)

24) Where the full trial protocol can be accessed, if available

Does your paper address CONSORT subitem 24? *

Cite a Multimedia Appendix, other reference, or copy and paste relevant sections from the manuscript (include quotes in quotation marks "like this" to indicate direct quotes from your manuscript), or elaborate on this item by providing additional information not in the ms, or briefly explain why the item is not applicable/relevant for your study

Full trial protocol was not available

25) Sources of funding and other support (such as supply of drugs), role of funders

Does your paper address CONSORT subitem 25? *

Copy and paste relevant sections from the manuscript (include quotes in quotation marks "like this" to indicate direct quotes from your manuscript), or elaborate on this item by providing additional information not in the ms, or briefly explain why the item is not applicable/relevant for your study

Funding: This research was funded by IRSC Catayseur (grant number: CRS-2021-118), Services et recherches psychiatriques AD, and Lévesque Foundation.

X27) Conflicts of Interest (not a CONSORT item)

X27-i) State the relation of the study team towards the system being evaluated

In addition to the usual declaration of interests (financial or otherwise), also state the relation of the study team towards the system being evaluated, i.e., state if the authors/evaluators are distinct from or identical with the developers/sponsors of the intervention.

	1	2	3	4	5	
subitem not at all important	○	○	○	○	○	essential

Does your paper address subitem X27-i?

Copy and paste relevant sections from the manuscript (include quotes in quotation marks "like this" to indicate direct quotes from your manuscript), or elaborate on this item by providing additional information not in the ms, or briefly explain why the item is not applicable/relevant for your study

"Conflicts of Interest None declared"

About the CONSORT EHEALTH checklist

As a result of using this checklist, did you make changes in your manuscript? *

- ○ yes, major changes
- ◉ yes, minor changes
- ○ no

What were the most important changes you made as a result of using this checklist?

Votre réponse

How much time did you spend on going through the checklist INCLUDING making changes in your manuscript *

more than 6 hours

As a result of using this checklist, do you think your manuscript has improved? *

- ○ yes
- ◉ no
- ○ Autre :

Would you like to become involved in the CONSORT EHEALTH group?

This would involve for example becoming involved in participating in a workshop and writing an "Explanation and Elaboration" document

- ○ yes
- ◉ no
- ○ Autre :

Effacer la sélection

Any other comments or questions on CONSORT EHEALTH

Votre réponse

STOP - Save this form as PDF before you click submit

To generate a record that you filled in this form, we recommend to generate a PDF of this page (on a Mac, simply select "print" and then select "print as PDF") before you submit it.

When you submit your (revised) paper to JMIR, please upload the PDF as supplementary file.

Don't worry if some text in the textboxes is cut off, as we still have the complete information in our database. Thank you!

Final step: Click submit !

Click submit so we have your answers in our database!

Envoyer Effacer le formulaire

N'envoyez jamais de mots de passe via Google Forms.

Ce contenu n'est ni rédigé, ni cautionné par Google. Signaler un cas d'utilisation abusive - Conditions d'utilisation - Règles de confidentialité

Google Forms
